# Signaling pathways in obesity: mechanisms and therapeutic interventions

**DOI:** 10.1038/s41392-022-01149-x

**Published:** 2022-08-28

**Authors:** Xue Wen, Bohan Zhang, Beiyi Wu, Haitao Xiao, Zehua Li, Ruoyu Li, Xuewen Xu, Tao Li

**Affiliations:** 1 Department of Plastic and Burn Surgery, National Clinical Research Center for Geriatrics, West China Hospital of Sichuan University, Chengdu, 610041 China; 2 Laboratory of Mitochondria and Metabolism, West China Hospital of Sichuan University, Chengdu, 610041 China; 3 Department of Anesthesiology, National-Local Joint Engineering Research Centre of Translational Medicine of Anesthesiology, West China Hospital of Sichuan University, Chengdu, 610041 China

**Keywords:** Metabolic disorders, Molecular medicine

## Abstract

Obesity is a complex, chronic disease and global public health challenge. Characterized by excessive fat accumulation in the body, obesity sharply increases the risk of several diseases, such as type 2 diabetes, cardiovascular disease, and nonalcoholic fatty liver disease, and is linked to lower life expectancy. Although lifestyle intervention (diet and exercise) has remarkable effects on weight management, achieving long-term success at weight loss is extremely challenging, and the prevalence of obesity continues to rise worldwide. Over the past decades, the pathophysiology of obesity has been extensively investigated, and an increasing number of signal transduction pathways have been implicated in obesity, making it possible to fight obesity in a more effective and precise way. In this review, we summarize recent advances in the pathogenesis of obesity from both experimental and clinical studies, focusing on signaling pathways and their roles in the regulation of food intake, glucose homeostasis, adipogenesis, thermogenesis, and chronic inflammation. We also discuss the current anti-obesity drugs, as well as weight loss compounds in clinical trials, that target these signals. The evolving knowledge of signaling transduction may shed light on the future direction of obesity research, as we move into a new era of precision medicine.

## Introduction

Obesity, defined as a body mass index (BMI) ≥30 kg/m^2^, is a complex chronic disease characterized by an excessive accumulation of fat or adipose tissue in the body.^1^ According to a report by the Non-Communicable Disease Risk Factor Collaboration, the prevalence of obesity increased worldwide from 1975 to 2016, ranging from 3.7% in Japan to 38.2% in the United States.^2^ The World Health Organization (WHO) describes obesity as one of the most blatantly visible and under-appreciated public health problems that increase the risk of multiple diseases, such as type 2 diabetes (T2D), cardiovascular disease, hypertension, nonalcoholic fatty liver disease, and certain cancers.^3–6^ Although the positive relationship between obesity and individual mortality/morbidity has been recognized for more than 20 years, the global prevalence of obesity continues to increase, and the WHO estimates that one out of five adults worldwide will be obese by 2025.^4^

Usually, obesity occurs when the body’s energy intake exceeds energy expenditure, which is influenced by inherited, physiological, and/or environmental factors.^7,8^ Indeed, genome-wide association studies have identified more than 300 single-nucleotide polymorphisms and 227 genetic variants related to obesity, although their functional impact on the obese phenotype is still a mystery.^9,10^ Accumulating evidence shows that unhealthy lifestyles lead to obesity.^11–14^ Moreover, exposure to environmental endocrine disruptors such as bisphenol A and perfluoroalkyl substances also increases susceptibility to obesity.^15–18^ Even worse, these acquired factors not only disturb the balance of energy metabolism at the posttranscriptional level,^19^ but also change the epigenetic inheritance of individuals and thereby make their offspring more susceptible to obesity.^20–22^

With advances in science and technology as well as the rapid growth of the pharmaceutical industry, tremendous achievements have been made in the fight against obesity;^23–25^ several strategies, such as calorie restriction, lifestyle management, pharmacotherapy, and bariatric surgery, have been proposed as anti-obesity remedies.^26–29^ Nonetheless, these interventions are incapable of meeting the global magnitude of medical needs. Recently, numerous factors/signals involved in appetite regulation and peripheral energy absorption, storage, and consumption have been revealed.^30–32^ These progressions shed light on the understanding of the occurrence of obesity. Some compounds targeting these signals have been translated into clinical uses. For example, appetite regulation, a hotspot of anti-obesity research, is regulated by both the central melanocortin pathway and peripheral signals such as leptin and gut hormones. Glucagon-like peptide 1 (GLP-1), a gut-derived hormone capable of decreasing blood sugar levels and improving glucose tolerance by promoting insulin secretion through cyclic adenosine monophosphate (cAMP)-based signaling pathways,^33–35^ can also reduce appetite by directly stimulating proopiomelanocortin (POMC)/cocaine- and amphetamine-regulated transcript (CART) (anorexigenic neurons) but suppressing agouti-related protein (AgRP)/neuropeptide Y (NPY) neurons (orexigenic neurons) through γ-aminobutyric acid (GABA)-dependent signaling.^32^ These findings make GLP-1 a crucial target for the treatment of obesity and other metabolic disorders.^36–38^ Indeed, Liraglutide, a kind of GLP-1 analog, has been introduced into the clinical treatment of T2D and obesity.

**Fig. 1:**
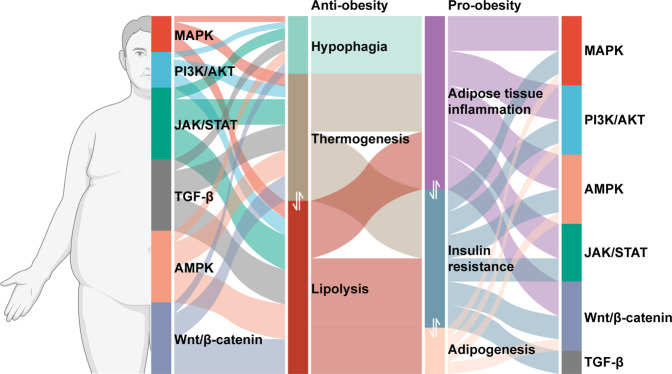
Signaling pathways involved in pro-obesity and anti-obesity mechanisms. Insulin resistance, adipose tissue inflammation, and adipogenesis constitute pro-obesity mechanism. Anti-obesity mechanism is composed of thermogenesis, lipolysis, and hypophagia

Although the underpinnings of its pathogenesis are not yet fully understood yet, obesity is well recognized as a heterogeneous disorder regulated by multiple pathways.^39–42^ The evolving understanding of the signaling pathways involved in obesity occurrence and development allows us to fight obesity in a more precise way. In this review, we summarize the signals/pathways involved in the pathogenesis of obesity, specifically in appetite regulation, adipose tissue metabolism and function, glucose hemostasis, and energy expenditure (Fig. 1), and discuss the current anti-obesity medications (AOMs) in clinical use or under clinical trials, that target these signals.

## Signaling pathways in the pathogenesis of obesity

### Obesity and the MAPK pathway

**Fig. 2:**
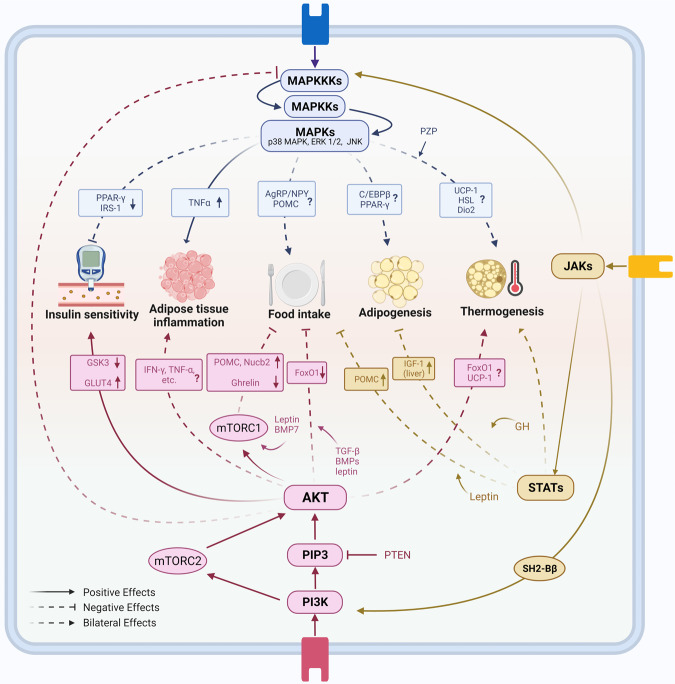
MAPK, PI3K, and JAK/STAT signaling pathways in obesity pathogenesis. MAPK signaling pathway includes a three-tiered kinase cascade consisting of MAPK kinase kinases (MAPKKKs), MAPK kinases (MAPKKs), and MAPKs. MAPKs such as ERK1/2, JNK, and p38 MAPK play complicated roles in adipogenesis and appetite regulation. Activation of MAPKs induced adipose tissue inflammation in obesity. MAPKs also cause insulin resistance in obesity by inactivating IRS1 directly and PPAR-γ indirectly. MAPKs signaling pathway plays diverse roles in adipose tissue browning and thermogenesis. PI3K-AKT pathway is closely related to insulin signaling. It increases GLUT4 and downregulates GSK3, resulting in insulin effects. PI3K-AKT signaling in lymphoid cells reduces adipose tissue inflammation to fight against obesity, while it results in the opposite direction in myeloid cells. Besides, PI3K-AKT-mTOR signaling negatively regulates food intake and has a bidirectional effect on thermogenesis. JAK-STAT signaling pathway consists of JAK1, 2, and 3, and STAT family includes STAT1, 2, 3, 4, 5a, 5b, and 6. JAKs cannot only activate STATs, but also MAPKKKs and PI3K. JAK-STAT pathway participates in leptin-mediated anorectic effects. In the liver, the activation of JAK-STAT signaling is negatively related to the accumulation of fat. Notably, there are different impacts from different JAKs and STATs on BAT-related thermogenesis

Mitogen-activated protein kinases (MAPKs) are critical mediators of signal transduction in mammalian cells.^43^ MAPK signaling contains a three-tiered kinase cascade composed of a MAPK kinase kinase (MAPKKK), a MAPK kinase (MAPKK), and the MAPK, which connects extracellular stimuli to intracellular signals.^44^ Upon phosphorylation by MAPK, downstream transcription factors are activated to mediate gene expression and initiate cellular events such as proliferation, inflammation, differentiation, and apoptosis.^45,46^ MAPK signaling members, including extracellular signal-regulated kinase (ERK) 1/2, c-Jun N-terminal kinase (JNK), and p38 MAPK, play a pivotal role in the regulation of appetite, adipogenesis, glucose homeostasis, and thermogenesis (Fig. 2).^47,48^

MAPK-mediated appetite regulation, as well as other MAPK functions in the central nervous system (CNS), contributes to the pathogenesis of obesity. ERK1/2 enhances glucose-stimulated POMC expression in hypothalamic neurons and participates in anorexigenic action.^49^ Moreover, JNK3 is essential in the effect of the leptin on AgRP neurons in high-fat diet (HFD)-fed mice.^50^ In addition, JNK1 knockout in the CNS decreases food intake and enhances energy expenditure by blocking the negative feedback of the hypothalamic-pituitary–thyroid axis, and ablation of JNK1 and JNK2 in the pituitary reduces the expression of Dio2, a negative regulator blocking thyroxine-mediated adaptive thermogenesis and lipid accumulation.^51,52^

ERK signaling is indispensable in the early steps of adipocyte differentiation, as ERK1^−/−^ mice are resistant to the development of adiposity under HFD feeding; preadipocytes from these mice as well as embryo fibroblasts exhibit impaired adipogenesis.^53^ However, there are in vitro studies with the opposite observation that sustained activation of ERK decreases adipogenesis by inhibiting peroxisome proliferator-activated receptor (PPAR)γ expression via MAPK-mediated phosphorylation.^54,55^ Considering the different experimental models, these controversial results should be interpreted cautiously. In vitro studies usually lack an appropriate microenvironment for cell interaction, and that may contribute to the inconsistency between in vitro and in vivo results. Similarly, the role of p38 MAPK in adipogenesis is also controversial. In primary embryonic fibroblasts from embryonic mice and preadipocytes from adulthood mice with p38 MAPK subunit knockout or inhibition, the phosphorylation of CCAAT-enhancer binding protein (C/EBP) β is enhanced, and PPARγ expression is increased, suggesting that p38 MAPK suppresses adipogenesis.^56^ Conversely, increased p38 MAPK activity is observed during human preadipocyte differentiation in vitro, and pharmacological inhibition of p38 MAPK in these cells reduces the accumulation of triglycerides and the expression of PPARγ together with other adipogenesis markers.^57^ Suppression of p38 MAPK activity also blocks adipogenesis in 3T3-L1 cells.^58^ In vivo, treatment with a p38 inhibitor reduces C/EBPβ phosphorylation and decreases PPARγ expression.^59^ In human white adipose tissue (WAT), the increased number of hypertrophic adipocytes is also associated with the upregulated p38 MAPK signals, and the phosphorylated p38 MARK is coupled with fasting levels of triglycerides, insulin, and glucose.^60^ Together, these findings suggest that p38 MAPK has bifunctional effects on adipocyte differentiation and adipogenesis. There is a possible interpretation that p38 MAPK functions differently in human and mouse preadipocytes.

There is a complex association between obesity and insulin resistance. The MAPK signaling pathway is closely involved in the development of insulin resistance. By dephosphorylating and deactivating multiple MAPKs, dual specificity phosphatase 9 restores the tyrosine phosphorylation level of insulin receptor substrate-1 (IRS1) and its capacity to mediate insulin signal transduction.^61^ Similarly, deficiency of caspase recruitment domain 9, an endogenous activator of MAPKs, mitigates HFD-induced insulin resistance and adipocyte enlargement.^62^ Phosphorylation of PPARγ by ERK enhances the ability of transcriptional coactivator with PDZ-binding motif to negatively regulate PPARγ and impair insulin sensitivity.^63^ JNK1 and JNK2 induce insulin resistance via serine/threonine phosphorylation of IRS, while JNK3 may improve insulin sensitivity in obesity.^64^ Ablation of MAPK phosphatase-1 in skeletal muscle, which activates both JNK and p38 MAPK, leads to increased insulin sensitivity and elevated energy expenditure, making mice resistant to the development of diet-induced obesity.^65^ However, the p38 MAPK pathway, through enhancement of the mRNA stability and nuclear migration of X-box binding protein 1 in the liver, maintains glucose homeostasis in the context of obesity, demonstrating its complicated impacts in different models.^66^ A recent study summarized that regulation of lipid metabolism by p38 MAPK was tightly connected to calcium ions.^67^ Notably, insulin resistance in adipose tissue may result from the chronic inflammation induced under obese condition. Inhibition of MAPKs is associated with less inflammatory cell infiltration, improved glucose tolerance, and ameliorated adipocyte enlargement.^62^ In adipose tissues from HFD-fed mice, integrated multiomic analysis shows that the inflammatory genes are enriched in MAPK pathways in macrophages.^68^ Licochalcone F, a synthetic retrochalcone, was found to inhibit tumor necrosis factor (TNF)α-induced expression of inflammatory factors and further alleviated glucose tolerance, reduced adipocyte size, and decreased macrophage infiltration in WAT, by interacting with MAPK signaling pathway.^69^

Brown adipose tissue (BAT) thermogenesis alleviates obesity by increasing energy expenditure. This process is regulated by MAPK signaling.^70–72^ Thermogenic gene expression stimulated by substances including IL-27, irisin, cinnamaldehyde, and withaferin A, is perturbed by p38 MAPK or ERK inhibitors.^70,73–75^ Overexpression of mitogen-activated protein kinase kinase 6 (MEK6), an upstream repressive factor of p38/ERK, decreases the expression of uncoupling protein 1 (UCP1) and hormone-sensitive lipase (HSL) in adipocytes.^76^ Other stimuli, such as cold exposure, promote browning by inducing p38 MAPK signaling and secretion of fibroblast growth factor (FGF)21.^77^ Interestingly, pregnancy zone protein, a novel hepatokine identified in the context of intermittent fasting, can promote p38 MAPK-dependent UCP1 expression in BAT, exhibiting therapeutic potential in the treatment of obesity.^78^

### Obesity and the PI3K/AKT pathway

The phosphatidylinositol 3-kinase (PI3K)/AKT signaling pathway is a key regulator of cell growth and proliferation, and aberrant activation of this pathway promotes the development of obesity.^79–81^ PI3K and AKT are two major nodes in this pathway, which are activated by upstream signals such as hormones and growth factors. Upon activation, PI3K converts phosphatidylinositol 4,5-bisphosphate (PIP2) to phosphatidylinositol 3,4,5-trisphosphate (PIP3), activates phosphoinositide-dependent kinases and AKT,^82,83^ and then leverages glycogen synthase kinase (GSK)3, PKCs, and the forkhead box (Fox) family to regulate glycogen synthesis, glucose uptake, and adipogenesis, respectively (Fig. 2).^84,85^ Mammalian target of rapamycin (mTOR) is one of the key downstream targets of PI3K/AKT pathway, referred to as PI3K/AKT/mTOR pathway together with the upstream sometimes. mTOR forms two distinct complexes, mTORC1 and mTORC2; raptor and PRAS40 are the specific subunits of mTORC1, whereas rictor, mSIN1, and Protor1/2 are the specific subunits of the mTORC2 complex.^86,87^ mTORC1 and mTORC2 act differently in the PI3K/AKT/mTOR signaling pathway and both are closely associated with the pathogenesis of obesity (Fig. 2).

The PI3K/AKT pathway regulates appetite via the CNS and peripheral tissues. It has been reported that leptin acts on the mediobasal part of the hypothalamus to suppress food intake partially through PI3K-AKT-FoxO1 pathway,^88^ and selective inhibition of PI3K abolishes the effect of leptin.^89^ mTOR also contributes to appetite regulation in the central and peripheral systems. Stimulation of mTOR in the hypothalamus decreases food intake and ameliorates age-dependent obesity in animal studies by activating POMC neurons.^88,90,91^ Transforming growth factor (TGF)-β/bone morphogenetic proteins (BMPs) in the hypothalamus closely interact with PI3K/AKT pathway to reduce appetite and mitigate obesity.^92^ A study showed that intracerebroventricular administration of BMP7 has an anorectic effect, which could be completely abolished by rapamycin pretreatment, indicating the existence of leptin-independent BMP7-mTOR-p70S6K signaling.^93^ In peripheral tissues, stimulation of mTOR in gastric X/A-like cells decreases the production of ghrelin, an orexigenic hormone that also decreases UCP1 expression.^94,95^ Similarly, secretion of Nucb2/nesfatin1, another hormone with anorexigenic effects, is enhanced by activation of mTORC.^96^

The PI3K/AKT pathway is indispensable to the insulin signaling pathway. Dysregulation of this signaling is associated with the severity of obesity and insulin resistance.^97–99^ Negative correlation between AKT activity and body fat percentage has been found both in animal models and humans, and AKT may be responsible for insulin resistance in the obese population.^100,101^ Inhibition of PI3K/AKT signaling leads to degradation of Sort1, an element of the glucose transporter 4 (GLUT4) storage vesicles, and decreases insulin sensitivity.^102,103^ Similar findings were obtained in mice with overexpressed phosphotyrosine interaction domain containing 1, which impairs PI3K/AKT signaling and directly interacts with low-density lipoprotein receptor-related protein (LRP)1, another part of GLUT4 vesicles.^104^ Furthermore, repression of PPARγ, the key regulator of adipocyte differentiation, leads to insulin resistance via PI3K/AKT signaling.^105^ However, it is plausible that manipulation of the PI3K/AKT pathway can regulate early adipogenesis. In support of this point, alchemilla monticola functions its anti-adipogenic effect via inhibiting this pathway.^106^ As a negative regulator of PI3K-mediated signal transduction, phosphatase and tensin homolog (PTEN) can also impact insulin effects. Metformin was reported to restore insulin resistance via 5′-AMP-activated protein kinase (AMPK)-mediated downregulation of PTEN.^107^ Notably, loss of PTEN could lead to obesity with preserved insulin sensitivity.^108^ PTEN haploinsufficiency in humans increases the risk of obesity as a monogenic factor but decreases the risk of T2D because of enhanced insulin sensitivity.^109^ As the largest insulin-sensitive organ, skeletal muscle has a significant role in glucose and lipid homeostasis. In the muscle of ob/ob mice, the expression of AKT2 was lower, and insulin resistance was observed in vitro.^110^ The PI3K inhibitor wortmannin fully inhibits insulin-stimulated glucose uptake in skeletal muscle.^111^ RalGAPα1 mainly exists in skeletal muscle, blunts insulin effects by preventing translocation of GLUT4, and can be inactivated by AKT. When blocking the inactivation process of RalGAPα1 by AKT, mice showed greater fat mass, larger body weight, and elevated levels of lipid in the bloodstream in adulthood.^112^ Another important organ, liver, also participates in glucose and lipid metabolism. PI3K/AKT/mTOR and PI3K/AKT/FoxO1 pathways in hepatocytes are parts of insulin signaling, and participate in hepatic glucose and lipid metabolism, such as de novo lipogenesis (DNL) and hepatic glucose production (HGP).^113^ Using specific knockout mice, Titchenell et al. demonstrated that activation of both of the above signaling pathways by insulin was necessary and sufficient for insulin-mediated lipid metabolism in the liver. They also found that PI3K/AKT/FoxO1 pathway contributes to insulin-mediated suppression of HGP.^114^ GSK3 is one of the substrates of AKT. Proteomics and phosphoproteome analysis revealed a downregulated substrate motif of AKT and hyperactivation of GSK3 in islets of obese diabetic mice, with the latter at least partly contributing to β cell failure.^115^ Intriguingly, mice carrying mutant GSK3, which blocks phosphorylation by AKT, have higher energy expenditure and are protected from HFD-induced metabolic syndrome.^116^ Some microRNAs, such as miR-33, miR-143, and miR-153, can inhibit the activity of the PI3K/AKT pathway and induce glucose intolerance in obesity.^117^

Hyperinsulinemia is both the cause and the consequence of insulin resistance.^118^ The activation of PI3K and phosphorylation of AKT are blunted in human myoblasts under continuous high insulin exposure.^119^ PI3K is also inhibited by the activation of glucocorticoid receptor, which contributes to insulin resistance in Cushing’s syndrome.^120^ Adipose tissue inflammation is another cause of impaired insulin tolerance. CD4+ T cells regulate inflammation in adipose tissue and obesity. A recent study identified Kruppel-like zinc-finger family 10 in CD4+ T cells as an essential regulator of obesity, insulin resistance, and fatty liver, the effects of which are mediated by PI3K-AKT-mTOR signaling.^121^ Conversely, specific ablation of the insulin receptor in myeloid cells led to reduced obesity-associated inflammation in adipose tissue.^122^ These opposite results indicate the different roles of PI3K/AKT signaling in lymphoid and myeloid cells.

In addition, mTORC1-p70 ribosomal S6 kinase 1 (S6K1) plays an essential role in insulin action. It is upregulated and has a positive correlation with insulin resistance in human visceral fat tissue.^123^ Furthermore, deficiency of this signaling results in less adipose tissue mass and enhanced lipolysis.^124^ The dedicator of cytokinesis 5 is widely expressed in vivo and reinforces insulin sensitivity by inhibiting mTORC1-S6K1.^125^ On the other hand, mTORC2 is essential in insulin-inhibited hepatic gluconeogenesis, and long-term rapamycin administration impairs insulin sensitivity by disrupting mTORC2 function.^126^ Whereas classic PI3K/AKT signaling activates mTOR, the subclasses of PI3K, including class II and class III, play different roles in the regulation of mTOR and glycerolipid metabolism. PI3KC2β in class II PI3K and its derivative, PtdIns-(3,4)-P2, promote the interaction between endosomes/lysosomes and mTOR1 and inhibit mTORC1, and class III PI3K stimulates mTORC1 in multiple ways to influence the effects of insulin.^127,128^

The PI3K/AKT pathway also plays a role in thermogenesis.^129^ HFD feeding induces the expression of the signaling scaffolding protein Gab2 in adipose tissues. Deletion of Gab2 in mice increases the expression of UCP1 and other thermogenic genes in BAT and attenuates HFD-related weight gain through downregulation of the PI3K-Akt-FoxO1 signaling pathway.^130^ Whole-body overexpression of PTEN, which counteracts PI3K-mediated signal transduction, activates BAT, decreases body weight, and increases appetite in mice.^131^ In contrast, PTEN knockout in hypothalamic leptin-sensitive neurons increases PI3K activity and leads to browning of WAT and weight loss.^132^ A possible explanation is that systemic overexpression of PTEN exerts opposing effects in both the central and peripheral systems, but is more potent in the latter. Notably, upregulation of UCP1 expression by albiflorin is attributed to the activation of AMPK and PI3K/AKT pathways because the effect could be eliminated when cells were cotreated with the AMPK inhibitor Compound C or the PI3K inhibitor LY294002.^133^ Through the PI3K/AKT pathway, glutamine supplementation reduces waist circumference in overweight volunteers and improves glucose homeostasis in the adipose mass of HFD-fed rats.^134^ Suppression of mTORC1 in BAT, by ablation of raptor or dissociation of raptor by growth factor receptor binding protein-10, enhances mitochondrial respiration and thermogenesis, suggesting that mTORC1 per se has a negative effect on energy expenditure.^135–137^ Meanwhile, mTORC1 is also indispensable for β-adrenergic stimulation-induced brown adipogenesis under cold exposure through the phosphorylation of S6K1 to promote protein synthesis.^138,139^ Similarly, reducing the expression of β-adrenergic receptors via the response gene to complement 32 lowers mTORC1/S6K1 activity and decreases thermogenic gene expression.^140^ mTORC2 reduces UCP1 expression in BAT, and ablation of rictor, an essential component of mTORC2, increases thermogenesis and alleviates HFD-induced obesity through the Sirtuin 6 (Sirt6)-FoxO1 pathway.^141^

### Obesity and the JAK/STAT pathway

The Janus kinase (JAK)/signal transducer and activator of transcription (STAT) pathway is one of the major intracellular signal transduction pathways and is an essential downstream mediator for various cytokines, hormones, and growth factors. The whole family of STAT proteins (STAT1, 2, 3, 4, 5a, 5b, and 6) can be activated by tyrosine phosphorylation in response to cytokine and growth factor stimulation.^142^ The binding of cytokines or growth factors to their cognate receptors activates JAKs (JAK1, JAK2, JAK3, or Tyk2), enabling them to transphosphorylate each other and the cytoplasmic tail of the receptor on tyrosine residues.^143,144^ The receptor subunits then provide a docking site for STAT proteins, which are in turn phosphorylated as well.^142^ The phosphorylated STAT proteins translocate to the nucleus, bind to specific DNA elements and regulate the transcription of targeted genes.^145^ The dysregulation of the JAK/STAT signaling pathway contributes to obesity directly or by interacting with other signaling pathways including MAPK and PI3K (Fig. 2).

The JAK/STAT signaling pathway is correlated with the melanocortin pathway since the energy homeostasis regulated by leptin is mediated by JAK/STAT.^146^ During leptin signaling, leptin receptor (LEPR), expressed at the plasma membrane as a dimer, activates receptor-associated JAK2 to phosphorylate LEPR, which then binds to STAT3 and STAT5. They are then phosphorylated by JAK2 to function as transcription factors.^147,148^ Activation of STAT3/STAT5 by LEPR is essential to control food intake.^149–152^ In addition, phosphorylated STAT3 induces the expression of suppressor of cytokine signaling 3, which acts as a feedback inhibitor of the leptin signaling pathway.^153^ Binding of leptin to LEPR results in downstream activation of Rho-kinase 1, which phosphorylates and activates JAK2 to maintain energy homeostasis.^154^ The binding also leads to JAK2 interaction with SH2-Bβ, which in turn promotes IRS1- and IRS2-mediated activation of the PI3K pathway.^155,156^ Then, it promotes transcription of POMC and increases the expression of carboxypeptidase with increased processing of POMC to α-melanocyte-stimulating hormone (α-MSH), and suppresses food intake.^157^ In contrast, suppression of JAK/STAT signaling in CNS is associated with decreased leptin sensitivity in POMC neurons.^158^

The accumulation of fat in the liver (hepatic steatosis) is a feature of obesity.^159^ This process is regulated in part through JAK/STAT signaling pathway by growth factors and cytokines.^160,161^ Studies have consistently suggested that hepatocyte-specific deficiency of STAT3 leads to insulin resistance and increased expression of gluconeogenic genes.^162–164^ Conversely, STAT3 activation in hepatocytes may prevent steatosis. Treatment of obese mice with STAT3-inducing cytokines (IL-6 and IL-22) or overexpression of STAT3 ameliorates hepatic fat accumulation.^165,166^ The pivotal role of the hepatic growth factor–JAK2–STAT5–IGF1 axis in lipid metabolism has been confirmed. Through activation of JAK2 and STAT5, growth factor plays a key role in the production of hepatic IGF1. The precise mechanism by which low growth factor levels contribute to obesity is controversial but may be attributed to decreased lipolysis in adipose tissue and increased hepatic steatosis.^167^ Loss of STAT5 signaling results in concurrent activation of STAT1 and STAT3 and intracellular lipid accumulation. Furthermore, there is evidence showing that mice with hepatocyte-specific deletion of JAK2 develop spontaneous steatosis as early as 2 weeks of age but manifest protection against HFD-induced insulin resistance and glucose intolerance.^168^

Peripheral JAK/STAT signaling pathway can be also activated by leptin.^169,170^ For instance, HFD-induced leptin secretion in adipose tissue increases the expression of the STAT3 target gene encoding caveolin-1, which decreases leptin signaling in a negative feedback manner.^171^ To further explore the role of STAT3 in adipocytes, Cernkovich et al. utilized an adipocyte-specific STAT3 mouse colony and observed increased body weight and adipose tissue mass with adipocyte hypertrophy, suggesting that STAT3 promotes lipolysis and inhibits adipogenesis.^172^ Moreover, mice lacking Tyk2 become progressively obese due to defective differentiation of BAT, indicating that the activation of STAT3 by Tyk2 is essential for BAT function.^173^ STAT4 also contributes to obesity-related pathophysiology by reducing insulin sensitivity and increasing adipocyte inflammation.^174^ Similarly, elevated interferon-γ levels and JAK–STAT1 signaling in obesity also lead to adipocyte dysfunction and insulin resistance.^175,176^ As the major upstream kinases required for STAT activity, JAK proteins also impact adipose function. Adipocyte-specific knockout of JAK2 in mice drives adiposity due to defective lipolysis,^177^ while pharmacological inhibition of JAK/STAT promotes UCP1 expression and browning of human adipocytes in vitro.^178^

### Obesity and the TGF-β signaling pathway

**Fig. 3:**
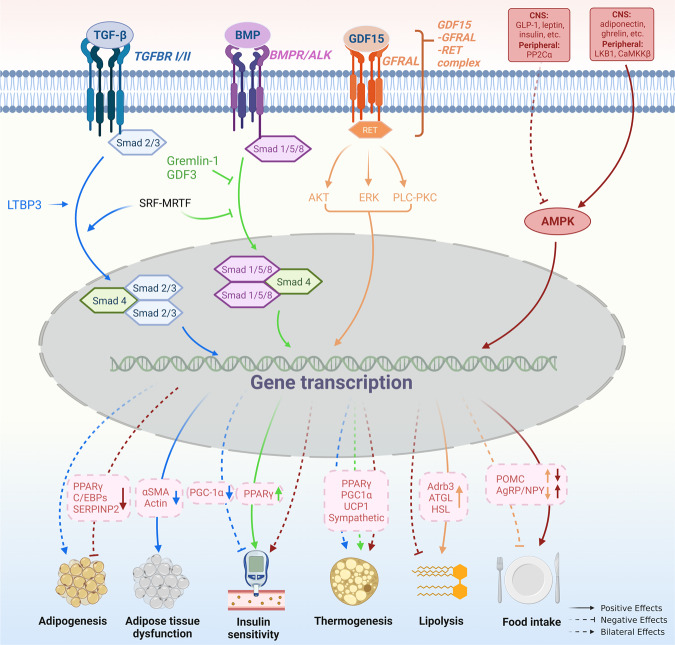
TGF-β and AMPK signaling pathways in obesity pathogenesis. The TGF-β superfamily consists of TGF-β1-3, GDFs, BMPs, etc., which play a diverse role in the development of obesity. TGF-β shows dual effects on adipogenesis/adipocyte differentiation. TGF-β inhibits MSC adipocyte commitment by phosphorylating and suppressing PPARγ and C/EBPs expression, through Smad3 signaling. However, pulsed TGF-β1 administration during the commitment phase shows a promotion effect on adipogenesis in MSC by down-regulating SERPINB2 expression. In adipocytes, TGF-β signaling is involved in adipose tissue dysfunction by enhancing the expression of myofibroblast signature genes. The role of TGF-β in BAT-associated thermogenesis is also controversial. Activation of TGF-β signaling by LTBP3 promotes WAT browning by modulating UCP1 expression, while hepatic TGF-β signaling contributes to HFD-induced steatosis and obesity by reducing mitochondrial respiration and inhibiting white-to-beige fat conversion. In addition, SRF - MRTF - axis which transcriptionally enhances the TGF-β but attenuates BMP signaling pathway suppresses brown adipogenesis. TGF-β/Smad3 signaling also plays a negative role in insulin sensitivity by suppressing PGC-1α expression in adipose tissue. BMP seems to play a contrary role to TGF- β in the regulation of insulin sensitivity by up-regulating PPARγ expression. Similar to TGF- β, the role of BMP in BAT-associated thermogenesis is inconsistent. BMP4 promotes WAT browning and this process is inhibited by Gremlin-1. However, BMP-4 signaling during the terminal differentiation phase can impair the acquisition of a mature brown adipocyte phenotype. GDF15, another member of TGF- β superfamily, was identified as a potential target for the treatment of obesity. By interacting with GFRAL and followed by the activation of AKT-, ERK-, and PLC-PKC signaling pathway, GDF15 stimulates lipolysis by up-regulating Adrb3, ATGL, and HSL expressions. It also inhibits food intake in a CNS-dependent manner via an unknown mechanism. AMPK is a heterotrimer complex. It is activated by adiponectin, ghrelin, etc. in CNS and LKB1 and CaMKKβ in peripheral tissue, and inactivated by GLP-1, leptin, etc. in CNS and PP2Cα in peripheral tissue. Activation of AMPK in CNS results in hyperphagia, insulin resistance, decreased thermogenesis, and weight gain. While, in adipocytes, it results in inhibited adipogenesis, insulin sensitiveness, enhanced thermogenesis, and weight loss. However, AMPK limits lipolysis since AMPK is an enzyme in case of energy shortage

The TGF-β superfamily consists of TGF-β1-3, activins/inhibins, growth differentiation factors (GDFs), myostatin, and BMPs, playing diverse roles in appetite regulation, lipid metabolism, and glucose homeostasis (Fig. 3).^179,180^

GDF15, a member of the TGF-β superfamily, has been identified as a central regulator of appetite and a potential target for the treatment of obesity.^181–183^ Mice lacking GDF15 exhibit obesity and pharmacological GDF15 administration to mice triggers a taste aversive response, suggesting that GDF15 plays a regulatory role in energy balance.^184^ Intracerebroventricular injection of GDF15 into the lateral ventricle of mice results in reduced food intake, and this effect requires an intact brainstem area postrema (AP) and nucleus of the solitary tract, suggesting that CNS is one of the targets of GDF15 action.^182^ Mechanistically, by interaction with glial cell line-derived neurotrophic factor (GDNF)-family receptor α-like (GFRAL) expressed in the brainstem, GDF15 decreases vagal sympathetic nervous system (SNS) activity (vagal efferent) and delays gastric emptying.^185^ In addition, hGDF-15-expressing xenografts show upregulated lipolytic genes (adrenoceptor beta 3, or Adrb3; adipose triglyceride lipase, or ATGL; HSL) in both WAT and BAT, resulting in decreased adipose tissue mass.^186^

TGF-β signaling shows dual effects on adipogenesis/adipocyte differentiation. A study by Ahdjoudj et al. found that TGF-β functioned to inhibit mesenchymal stem cell (MSC) adipocyte commitment by phosphorylating and suppressing PPARγ expression as well as the expression of C/EBPs, partially through mothers against decapentaplegic 3 (Smad3) signaling.^187,188^ Deletion of TGF-β receptor 2 in MSCs resulted in a marked increase in adipocyte expansion in murine bone marrow, which was accompanied by an increase in PPARγ expression.^189^ However, another study found that continuous TGF-β1 treatment enhanced osteoblast differentiation as evidenced by increased mineralized matrix production, while pulsed TGF-β1 administration during the commitment phase increased mature lipid-filled adipocyte numbers.^190^ Global gene expression analysis revealed that serpin peptidase inhibitor clade B (ovalbumin) member 2 (SERPINB2) was significantly downregulated in TGF-β1-treated cells, and silencing of SERPINB2 in untreated cells enhanced the adipogenic differentiation capacity of both marrow osteoblast and adipocyte progenitor cells.^190^ These results suggest that the function of TGF-β in adipogenesis is determined by the mode of administration, and SERPINB2 was identified as the TGF-β1-responsive gene through which it negatively regulates adipogenic differentiation. In adipocytes, TGF-β1 was proven to be involved in obesity-related adipose tissue dysfunction. Adipocytes from HFD-fed mice showed enriched TGF-β1 effector protein Smad at HFD-induced promoters and enhancers and were associated with myofibroblast signature genes.^191^

Plasma levels of TGF-β1 are elevated in noninsulin-dependent diabetes mellitus.^192^ TGF-β signaling regulates glucose tolerance and energy homeostasis, and systemic blockade of TGF-β/Smad3 signaling protects mice from obesity, diabetes, and hepatic steatosis by enhancing PPARγ coactivator 1α (PGC-1α) expression in adipose tissue.^193^ In addition, recent studies have reported that aerobic exercise can inhibit TGF-β to improve insulin resistance,^194^ and inhibition of TGF-β/Smad3 signaling can prevent β-cell apoptosis,^195^ which is indicative of the therapeutic potential of TGF-β/Smad3 antagonists in restoring insulin sensitivity and β-cell homeostasis in diabetes. BMP signaling also interacts with the insulin signaling system to coordinately regulate glucose homeostasis. BMP-2 and BMP-6 enhance insulin-mediated glucose uptake in both insulin-sensitive and insulin-insensitive adipocytes.^196,197^ This function was achieved by inducing the expression and activation of PPARγ, which improves insulin sensitivity.^198–201^ In addition, another member of the TGF-β superfamily, GDF-3, has been shown to affect glucose uptake in vitro by limiting BMP signaling and inducing insulin resistance in vivo, and GDF-3 expression was associated with obesity-linked PPARγ S273 phosphorylation.^202^ From the above data, it seems that TGF-β plays a negative role in glucose homeostasis regulation, whereas BMP functions oppositely to improve insulin sensitivity.

Inconsistent results were observed in regard to the role of TGF-β in energy expenditure. Latent TGF-β-binding protein 3 (LTBP3), which regulates TGFβ activity by forming intracellular complexes with the TGF-β pro-peptide, has been demonstrated to promote WAT browning by modulating UCP1 expression and mitochondrial oxygen consumption through TGF-β2 signaling.^203^ However, hepatic TGF-β signaling was found to contribute to HFD-induced steatosis and obesity by reducing mitochondrial respiration and inhibiting white-to-beige fat conversion, effects that are mediated by hepatocyte-derived exosomal let-7b-5p.^204^ In addition, the serum response factor (SRF)–myelin-related transcription factor (MRTF) axis transcriptionally enhances TGF-β but attenuates the BMP signaling pathway and thus suppresses brown adipogenesis.^205^ These results indicate that the TGF-β family may play diverse roles in BAT regulation, which is determined not only by its upstream characteristics but also by its origination and the specific pathways activated.

BMP4, another member of the TGF-β superfamily, is secreted by differentiated preadipocytes and drives a beige/brown adipose phenotype in preadipocytes.^206^ Expression of BMP4 promotes adipocytes of WAT to present brown fat characteristics, leading to a reduction in adiposity and related metabolic disorders.^207^ This process can be inhibited by Gremlin-1, an extracellular antagonist of BMPs.^206^ Knockdown of Gremlin-1 or treatment with BMP4 during adipocyte differentiation induces a shift from a white to a brown-like phenotype.^206^ Thus BMP4 and its antagonist Gremlin-1 together constitute a feedback cascade to control adipogenic commitment and differentiation. Further study suggests that BMP7 has similar effects on the white-to-brown transition as BMP4 in primary human adipose stem cells.^208^ In contrast, there are also studies showing that BMP4 signaling during the terminal differentiation phase can instead impair the acquisition of a mature brown adipocyte phenotype, favoring a more white-like phenotype, and likewise, exposure of mature brown adipocytes to BMP4 induces a brown-to-white-like adipocyte shift.^209,210^ BMP8B is another important regulator of energy balance. BMP8B is expressed in both peripheral tissues including BAT and the hypothalamus. It functions peripherally to increase the response of BAT to adrenergic stimulation while acting centrally to increase sympathetic output to BAT. Bmp8b-KO mice exhibit impaired thermogenesis and reduced metabolic rate, causing weight gain despite hypophagia.^211–213^ It is worth noting that the effect of BMPs is dependent not only on their own levels but also on levels of cellular BMPs antagonists making the cells resistant to secreted BMPs.^206^ Several antagonists, such as GREM1, GREM2, and NOGGIN, are expressed in adipose tissue.^206^ GREMLIN-1 and NOGGIN, two powerful and secreted BMP4 inhibitors, were found to be markedly increased in adipose tissue in obesity, inhibiting BMP4-induced precursor cell commitment/differentiation and white to beige/brown adipocyte conversion.^206,214^ Thus, WAT becomes resistant to BMP4 action in obesity due to the increased secretion of these antagonists.

### Obesity and the AMPK pathway

AMPK is a heterotrimer complex consisting of a catalytic subunit α (α1, α2) and two regulatory subunits β (β1, β2) and γ (γ1, γ2, γ3) and is activated by phosphorylation of the α subunit at Thr172.^215^ AMPK functions as a “fuel gauge” to monitor cellular energy status and is highly conserved across all eukaryotic species.^215,216^ Growing evidence suggests that brain AMPK plays a pivotal role in the development of obesity by regulating feeding, insulin sensitivity, BAT thermogenesis, and browning of WAT (Fig. 3).^217^

Activation of AMPK in CNS results in weight gain. David Carling and Caroline Small groups first demonstrated that hypothalamic AMPK regulates feeding behavior.^218^ This seminal study found that in vivo administration of leptin decreased hypothalamic AMPK activity and reduced food intake, while in vivo administration of ghrelin stimulated hypothalamic AMPK activity and increased food intake.^218^ A parallel work in the same year by Barbara Kahn et al revealed that AMPK is highly expressed in many hypothalamic regions and regulation of hypothalamic AMPK is part of a feedback system to the physiological modulation of feeding.^219^ Therefore, refeeding diminishes but fasting boosts the AMPK activity in the hypothalamus.^218,219^ From a macro-perspective, activation and inhibition of hypothalamic AMPK increases and decreases body weight, respectively.^219^ This was subsequently validated by the weight monitoring of mice lacking AMPKα2 in POMC or AgRP neurons of the arcuate nucleus (ARC). POMCα2KO mice developed obesity while AgRPα2KO mice developed an age-dependent lean phenotype.^220^ AMPK inhibition in both the ARC and the VMH can cause severe and prolonged hypoglycaemia.^221,222^ In contrast, AMPK activation in the VMH can cause insulin resistance.^222^ Moreover, accumulating evidence supports that hypothalamic AMPK manages BAT thermogenesis via its modulation of the SNS.^215,217^ Targeted administration of triiodothyronine in the VMH of the hypothalamus leads to decreased AMPK activity, elevated SNS activity, increased BAT thermogenesis, and reduced weight.^223^ Besides, central administration of estradiol inactivates AMPK in the VMH of the hypothalamus, resulting in SNS-mediated activation of BAT thermogenesis and weight loss.^224^ Furthermore, Nogueiras et al found that central injection of liraglutide in mice resulted in weight loss independent of hypophagia. Instead, such reduced weight is caused by AMPK-mediated BAT thermogenesis and adipocyte browning in the VMH of the hypothalamus.^225^

Intriguingly, the activation of AMPK in adipocytes results in weight loss. First, activated AMPK in brown and beige adipocytes increased non-shivering thermogenesis and improved insulin sensitivity.^226^ Second, it is reported that reduced body weight and improved insulin sensitivity by a low-calorie diet or bariatric surgery are closely related to increased AMPK activation in adipose tissue.^226^ Third, AMPK activation diminishes adipogenesis in adipocytes via shutting down eIF2α-dependent translation, activating WNT/β-catenin and Pref-1/ERK1/2/SOX9 pathways, and downregulating adipogenic markers including C/EBPβ, PPARγ, C/EBPα, FAS, aP2 and SREBP-1c.^227–232^ Fourth, studies have also reported the importance of AMPK substrates in obesity. For instance, both human and mouse studies link a bona fide AMPK substrate TBC1D1 to the development of obesity.^233,234^ Wang and Chen groups introduced a knockin mutation that prevents the phosphorylation of TBC1D1 by activated AMPK and found that the knockin mice developed obesity on a normal chow diet. Mechanistically, blockade of TBC1D1 phosphorylation in adipocytes promotes insulin-like growth factor 1 (IGF1) secretion and consequently activates the IGF1R/Akt/mTOR pathway, which in turn induces the expressions of lipogenic genes, resulting in weight gain.^235^ AMPK is activated in the setting of enhanced lipolysis like exercise and fasting. However, in adipocytes, AMPK counterintuitively limits lipolysis since AMPK is an enzyme in case of energy shortage.^236^ This could be explained by the fact that lipolysis is very demanding for energy homeostasis and the accumulation of free fatty acids from lipolysis into adipocytes may be detrimental to the energy-producing process because they are well-known mitochondrial uncouplers.^236,237^ The inhibition of lipolysis by activated AMPK served as a feedback mechanism preventing excessive energy consumption.

### Obesity and the Wnt/β-catenin signaling pathway

**Fig. 4:**
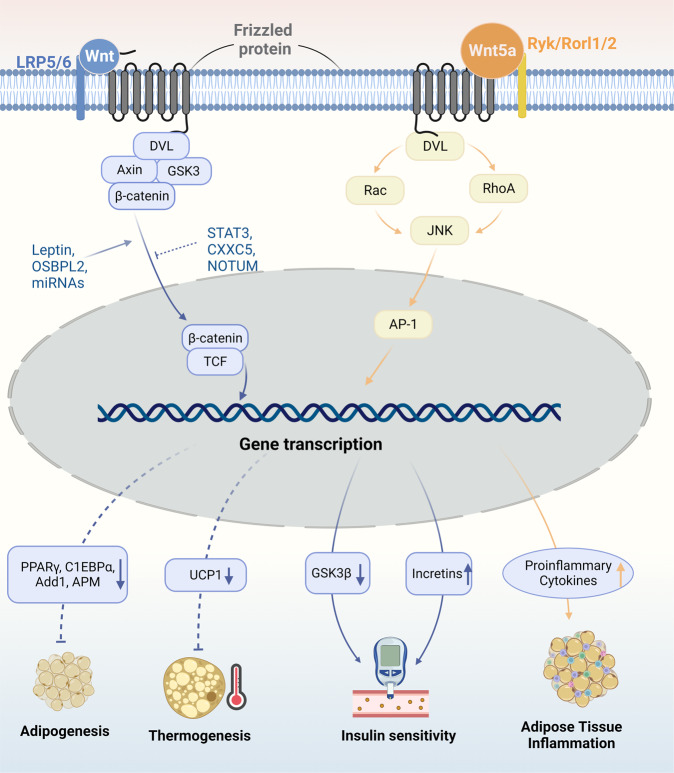
Wnt/β-catenin pathways in obesity pathogenesis. In the canonical Wnt pathway, upon activation by Wnt proteins, β-catenin is released and enters the nucleus as a transcription coactivator of TCF to regulate the transcription of target genes. The activation of Wnt/β-catenin pathway leads to, (1) the supersession of adipogenesis by down-regulating the expression of PPARγ, C1EBPα, Add1, APM, etc.; (2) the inhibition of BAT-related thermogenesis by down-regulating UCP-1; and (3) the increase of insulin sensitivity by down-regulating GSK3β expression in CNS while up-regulating incretins within the small intestinal epithelium. The canonical Wnt signaling can be stimulated by factors including leptin, OSBPL2, and miRNAs like miR-23b, miR-148b miR-4269, and miR-4429. It can also be inhibited by JAK/STAT3 pathway, CXXC5, and NOTUM. These factors are all involved in the pathogenesis of obesity by regulating Wnt/β-catenin signaling pathway. Additionally, Wnt5a, a part of the non-canonical Wnt pathway, induces obesity-associated inflammation in WAT in a JNK-dependent manner, which further contributes to the occurrence of insulin resistance in adipose tissue

The Wnt/β-catenin pathway is a canonical pathway in Wnt signaling and is composed of Wnt proteins, Frizzled and LRP5/6), Dishevelled proteins, Axin, GSK3, and β-catenin. In addition, there are two other noncanonical Wnt pathways, the Ca^2+^-dependent pathway, and the planar cell polarity pathway.^238,239^ The activation/inhibition of the Wnt signaling pathway leads to different effects in obesity pathogenesis, which is determined by the specific pathways of action (Fig. 4).

The Wnt/β-catenin pathway has been suggested to have a negative effect on adipogenesis and obesity.^240–243^ Wnt/β-catenin induces osteoblastogenesis from MSCs and simultaneously suppresses the expression of adipocyte-related genes including PPARγ and fatty acid synthase, thus inhibiting adipogenesis.^244,245^ Knockout of oxysterol-binding protein-like 2 (OSBPL2), a transport protein mediating the function of β-catenin, promoted the maturation of preadipocytes and caused an obese phenotype.^246^ When Wnt signaling was activated within adipose progenitor cells, mice showed significantly reduced visceral fat and a higher degree of fibrosis in subcutaneous WAT due to alternation of the adipocyte into a fibroblastic lineage.^247^ However, the stimulation of Wnt signaling within mature adipocytes did not yield the same result.^247^ Conversely, Wnt/β-catenin was found to be upregulated in mature adipocytes within WAT, and ablation of β-catenin in mature adipocytes exhibited resistance against HFD-induced adipose tissue expansion but not chow-diet adipose tissue.^248^ In another study, adipocyte-specific loss of β-catenin downregulated gene expression related to DNL and protected against HFD-induced obesity and metabolic dysfunction.^249^ Intriguingly, this study suggests that deficiency of β-catenin in adipocytes can be sensed and compensated for by CD45-/CD31- stromal cells to maintain tissue-wide Wnt signaling homeostasis in chow-fed mice, while with long-term HFD, this compensatory mechanism is overridden.^249^ Wntless, a chaperone protein for the secretion of Wnts, is essential for DNL in mature adipocytes and induced by HFD. Similarly, knockout of Wntless in adipose tissue did not lead to a lean phenotype under a chow diet because of compensation from surrounding stromal cells but reduced WAT mass in HFD-fed mice.^250^ Moreover, knockdown of LRP5, an essential protein in canonical Wnt signaling, in either abdominal or gluteal adipose progenitors leads to distinct biological outcomes: enhanced abdominal adipogenesis and suppressed gluteal adipogenesis.^251^ Therefore, Wnt/β-catenin signaling plays a complicated role in different fat depots, different diets, and different stages of adipogenesis.

The Wnt/β-catenin pathway influences insulin action and systemic glucose homeostasis.^252,253^ The canonical Wnt transcriptional effector TCF7L2 was found to be closely related to susceptibility to T2D.^254^ In visceral adipose tissues of patients with obesity-related diabetes and HDF-fed mice, the Wnt/β-catenin pathway is downregulated. Inhibition of CXXC-type zinc-finger protein 5 (CXXC5), a negative feedback regulator of Wnt signaling, alleviates the phenotype of obesity-related diabetes.^255^ Wnt signaling induces the synthesis of incretins within the small intestinal epithelium and is linked to T2D.^256^ In addition, Wnt5a, a part of the noncanonical Wnt pathway, has been proven to induce obesity-associated inflammation in WAT and contribute to dysregulation in glucose metabolism in a JNK-dependent manner.^257^

Wnt/β-catenin signaling contributes to the regulation of energy homeostasis.^258^ Wnt signaling was downregulated in leptin-deficient mice and this was rescued by leptin treatment.^259^ A recent study suggested that Wnt/β-catenin signaling mediates leptin effects by suppressing GSK3β, an inhibitor of insulin signaling.^260^ In addition, via integration of the leptin signal, Wnt/β-catenin signaling is associated with neuroendocrine regulation of body weight.^261^ Mice lacking β-catenin specifically in osteoblasts exhibit decreased fat accumulation and increased energy expenditure.^262^ Compared to lean controls, Wnt/β-catenin signaling in exosomes derived from obese visceral adipose tissue emerges as one of the top canonical pathways.^263^ Activation of Wnt/β-catenin signaling inhibits the browning of adipocytes,^264^ whereas suppression-enhanced browning is mainly displayed at early adipocyte differentiation, suggesting that Wnt/β-catenin-regulated browning is likely in beige precursor cells.^265^ Other organs, such as the liver, can promote the browning of WAT by secreting NOTUM, an inhibitor of Wnt signaling.^266^ The Wnt/β-catenin pathway is also responsible for STAT3-regulated preadipocyte differentiation, suggesting an interaction between the Wnt/β-catenin pathway and the JAK/STAT pathway during the early stage of adipogenesis.^267^

### Other signals/pathways

#### ER stress factors and the involved pathways

Endoplasmic reticulum (ER) is a critical organelle responsible for vital metabolic functions.^268^ ER stress refers to a condition in which unfolded or misfolded proteins accumulate in ER and leads to stress conditions.^269^ A plethora of evidence from animal and clinical studies shows that elevated ER stress in adipose tissue is induced by obesity, which in turn impairs ER functions and leads to metabolic dysfunction within the cell.^270^

**Fig. 5:**
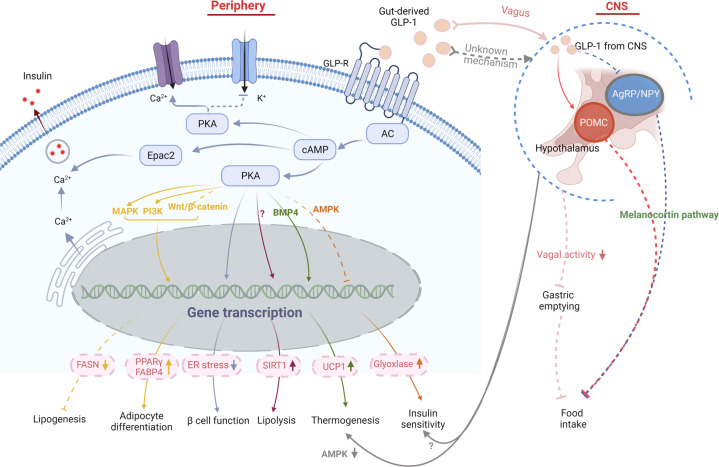
GLP-1 signaling pathway in obesity pathogenesis. The anti-obesity effect of GLP-1 can be mediated by either peripheral or central signals. In the periphery, the activation of GLP-R by gut-derived GLP-1 enhances the glucose-stimulated insulin secretion, through PKA-dependent or Epac2 pathway. By enhanced PKA activity, GLP-1 alleviates insulin resistance and leads to weight loss in obese diabetic mice by reducing ER stress and improving β-cell function. It also improves insulin sensitivity in peripheral tissue by suppressing AMPK-related pathway and elevating glyoxalase. By interacting with multiple signaling pathways including PI3K, MAPK, and Wnt4-β-catenin pathways, GLP-1 promotes pre-adipocyte differentiation by up-regulating PPARγ and FABP4, but suppresses lipogenesis in mature adipocytes by decreasing fatty acid synthase expression. GLP-1 also enhances lipolysis in WAT by increasing the expression and activity of Sirt1, through yet unknown mechanisms. Additionally, GLP-1 participates in the regulation of thermogenesis by inhibiting BMP4-related signaling pathway and thus induces the expression of thermogenic genes like UCP1. Gut-derived GLP-1 also interacts with GLP-R expressed in vagus, through which the information is transmitted upward to the CNS, which in turns suppresses vagal activity and gastric emptying, so as to increase satiety and reduce food intake. Besides, peripheral GLP-1 plays a role in the regulation of insulin sensitivity and BAT-related thermogenesis in a CNS-dependent manner. the latter is partially mediated by suppressing AMPK signaling pathway. Central GLP-1 produced by neurons in the caudal medulla is transmitted into the hypothalamus and functions to reduce food intake by activating POMC neurons while suppressing AgRP/NPY neurons in this area

Mesencephalic astrocyte-derived neurotrophic factor (MANF) is primarily retained in the ER under normal conditions. Under ER stress induced by inflammation or the accumulation of reactive oxygen species (ROS), MANF is released in large amounts into the cytoplasm and partially translocated into the nucleus. By activating the unfolded protein response (UPR) signaling cascade and negatively regulating nuclear factor kappa B (NF-κB) signaling, MANF inhibits the transcription of proinflammatory factors and improves ER homeostasis.^271^ MANF can also interact with multiple signaling molecules including p38, mTOR, AMPK, etc., via unknown mechanisms.^272^ Although the precise functions of MANF have not been fully clarified, emerging evidence supports that MANF is closely associated with the occurrence of obesity (Fig. 5).^273,274^ The regulatory role of MANF in energy homeostasis in the CNS and peripheral tissues seems to be discordant. MANF is abundantly expressed in the central neurons regulating appetite,^275^ and its expression in several hypothalamic nuclei that critically regulate food intake is likely to be affected by feeding state. Upon fasting, MANF expression in the hypothalamus of mice increased markedly. The upregulated MANF in the hypothalamus leads to the development of hyperphagia and obesity, while its reduction in the hypothalamus results in hypophagia and retarded body weight gain.^276^ Mechanistically, MANF induces the expression of PIP4k2b, an interacting partner of MANF in the ER, to trigger insulin resistance and disrupt insulin signaling in the CNS, leading to hyperphagia and fat mass accumulation.^276^ In contrast to the upregulation of MANF in the hypothalamus upon fasting, overnutrition leads to a decrease in MANF transcription in the subfornical organ, a forebrain sensory circumventricular organ controlling energy balance and hydration status.^277^ Although whether MANF also acts to positively regulate energy intake via the subfornical organ is unknown, the above evidence suggests that negative feedback may exist in the regulation of MANF expression patterns in the CNS via food intake. Peripherally, strong expression of MANF was observed in tissues and cells with high energy consumption, such as heart, muscle, and BAT.^275^ A recent study revealed that MANF is a feeding-induced hepatokine whose expression in the liver is strongly induced by HFD.^278^ Liver-specific MANF overexpression protected mice against HFD-induced obesity by promoting the browning of inguinal subcutaneous WAT.^278^ Mechanistically, MANF activates the p38 MAPK pathway to directly promote white adipocyte browning.^278^ Mice with MANF knockout in the liver showed impaired WAT browning and exacerbated diet-induced obesity, whereas subcutaneous injection of recombinant MANF retarded body weight gain in both diet-induced and genetic obese mouse models.^278^ These results indicate that peripheral MANF positively regulates thermogenesis and resists obesity. Of note, circulating MANF levels were found to be positively correlated with BMI in humans,^278^ indicating that obesity may increase the peripheral level of MANF in a compensator manner to relieve excessive weight gain. However, the exact role and mechanism of MANF in regulating energy balance still need further investigation, especially in regard to the different modes of action in the CNS and peripheral tissues.

Inositol-requiring enzyme 1α (IRE1α) is another evolutionarily conserved ER stress sensor that may serve as a critical switch governing energy balance.^279^ ER stress stimulates IRE1α oligomerization in ER membranes and autophosphorylation of IRE1α’s cytosolic domain.^279^ Activated IRE1α RNase catalyzes the unconventional splicing of Xbp1 mRNA and helps to generate a transcriptionally active transcription factor XBP1s to initiate the transcription of the key UPR gene to cope with ER stress.^280^ IRE1α can also function by interacting with TNF receptor-associated factor-2 and apoptosis signal-regulating kinase 1 to form a complex, which further activates downstream of stress kinases JNK and plays a crucial role in the regulatory machinery governing proteostasis and ER’s functional integrity.^281–283^ IRE1α can be activated by three major types of signals: nutrients, hormones, and immunological stimuli. Nutrients such as saturated fatty acids can activate IRE1α in a manner that does not rely on its unfolded protein-sensing ability.^284^ This, in turn, activates the NOD-like receptor thermal protein domain associated protein 3 inflammasome in macrophages and drives HFD-induced IL-1β secretion.^284^ Metabolic hormones such as insulin activate IRE1α–XBP1 pathway in livers as well as in primary hepatocytes and result in the enhanced de novo lipogenic program in an XBP1s-dependent manner.^285^ Some inflammatory stimuli including lipopolysaccharide (LPS) and IL-4, can also activate the Xbp1 mRNA-splicing activity of IRE1α by interacting with toll-like receptors (TLRs).^286^ The activation of IRE1α exerts a broad range of tissue- or cell-type-specific functions in energy metabolism. Centrally, IRE1α plays complex roles in appetite regulation. Mouse with exons 16 and 17 of gene encoding IRE1α deletion in POMC neurons shows marginal acceleration of HFD-induced obesity with considerable impairments in leptin and insulin sensitivity in POMC neurons and energy expenditure.^287^ In contrast, mouse with exon 2 fragment of IRE1 gene deletion in POMC neurons exhibits significant resistance to HFD-induced obesity and improvement of insulin resistance.^288^ In addition, increased energy expenditure and leptin sensitivity with higher production of α-MSH in the hypothalamus were also observed in mice with POMC neuron-specific ablation of IRE1α.^288^ Peripherally, mice with myeloid-specific IRE1α abrogation largely reversed HFD-induced M1-M2 imbalance in WAT and blocked HFD-induced obesity, insulin resistance, hyperlipidemia, and hepatic steatosis.^289^ In addition, myeloid-specific IRE1α abrogation increased WAT browning and energy expenditure in mice.^289^ These results suggest the multifaceted functions of IRE1α protein between CNS and periphery, and genetic deletion of different regions of IRE1α-encoding gene leads to apparent discrepancy in the phenotypes.

#### Immune-related pathways

Many of the comorbidities of obesity including T2D and cardiovascular disease are related to the dysimmunity induced by obesity.^290^ WAT is composed of various types of cells including adipocytes and immune cells.^291^ As an endocrine organ, WAT produces a variety of proinflammatory cytokines and integrates immune signaling in the dysfunctional metabolic status.^292^ Despite that the specific primordial trigger for sustained inflammation in obese WAT is unknown, this process is likely to be associated with metabolic stressors (from nucleic acids to lipids, from small compounds to macromolecules) arising from excessive adipocyte hypertrophy and hyperplasia induced by overnutrition, and also external stimuli such as the elevated levels of plasma LPS.^292^ Under these internal stressors and external stimuli, immune cells infiltrate and produce proinflammatory cytokines locally, resulting in WAT remodeling and insulin resistance. Mechanistically, obesity-related chronic inflammation in WAT is partially mediated, if not all, by TLRs expressed in adipocytes and macrophages.^293^ TLRs is an evolutionarily ancient family of pattern recognition receptors, which can recognize microbiological components such as the pathogen-associated molecular patterns (PAMPs) like LPS, and also internal stimuli such as nonesterified fatty acid.^294^ By activating TLR4/TLR2, WAT stressors or LPS stimulate NF-κB and JNK signaling, upregulate the expression of inflammatory cytokines including TNF-α and IL-6, and further induce insulin resistance in adipocytes and macrophages.^295,296^ TLRs-related pathways are also involved in the locally proinflammatory environment in BAT. The proinflammatory condition in BAT not only decreases the insulin sensitivity of BAT and impairs the uptake of fuel for thermogenesis, but also alters the activity of BAT by disturbing its energy expenditure mechanism. TLR2/4 were upregulated in the BAT from the obese mice, paralleled with the upregulation of inflammatory cytokines and chemokines in this tissue.^297^ Activation of TLR4 and TLR2 in brown adipocytes induces the activation of NF-κB and MAPK signaling pathways, leading to inflammatory cytokine/chemokine expression and attenuating both basal and isoproterenol-induced UCP1 expression.^297^ TLR4 activation by LPS also represses β3-adrenergic-mediated WAT browning and caused ROS production and mitochondrial dysfunction, whereas the deletion of TLR4 protects mitochondrial function and thermogenic activation.^298^

TLRs-related pathways are also involved in the regulation of the microorganism environment in the intestines.^299^ Given that the highest numbers of microbiomes are found in the gut, the role of gut microorganisms has been extensively studied and its polymorphism was implicated to be associated with obesity.^300^ Gut microbiological components play a crucial role in human metabolic regulation. With expressions of TLRs, colonocytes and endocrine cells are able to sense and transmit signals from PAMPs and thus functionally regulate inflammation, intestinal nutrient absorption, and insulin and incretins secretion.^299^ Activated TLRs mainly work through myeloid differentiation factor 88 protein (MyD88)-dependent and MyD88-independent signaling pathways.^301^ Animal study found that the deletion of MyD88 in intestines partially protects against diet-induced obesity, diabetes, and inflammation,^302,303^ indicating that the overactivation of MyD88 by some specific microbes may be one of the mechanisms of pathological gut microbial environment-related obesity.

Another pathway closely related to the inflammation status of obesity is the cyclic stimulator of the interferon genes (STING) signaling pathway. Usually, STING senses the presence of cytosolic DNA, either from the nucleus or mitochondria, and in turn, triggers downstream signaling to induce the expression of inflammatory and type I interferon genes in immune cells.^304^ Emerging evidence suggests that this signaling pathway may have additional functions beyond innate immune surveillance and may contribute to the chronic inflammation observed in obese patients (Fig. 3).^305–309^ Although the notion that obesity triggers chronic, low-grade inflammation has been recognized for decades, the pivotal role of the STING pathway in obesity has recently been appreciated.^310–312^ The STING pathway can be activated by palmitic acid, leading to mitochondrial damage and thereby mtDNA leakage. Through the cytosolic DNA sensor cGAS, mtDNA activates the STING-interferon regulatory Factor 3 pathway and induces a chronic sterile inflammatory response in mouse adipose tissue.^313,314^ In STING-deficient mice, the effects of diet-induced obesity, including endothelial inflammation (in adipose tissue), insulin resistance, and glucose intolerance, were alleviated.^314^ These findings support the notion that STING signaling plays a critical role in obesity-related adipose inflammation and insulin resistance. Of note, adipose tissue-specific knockout of DsbA-L, a chaperone-like protein identified in the mitochondrial matrix that maintains mitochondrial integrity, activates the cGAS-STING pathway in adipose tissue and exacerbates obesity-related pathology, while fat-specific overexpression of DsbA-L protected mice against HFD-induced activation of the STING pathway and chronic inflammation.^313^ These results suggest that maintaining mitochondrial homeostasis to target STING activation may be an alternative anti-obesity strategy. After translocation from the ER to the Golgi, STING can activate TANK-binding kinase 1 (TBK1), a downstream target that is essential for STING-dependent signaling.^315^ Recent studies report that systemic or adipocyte-specific TBK1 knockout attenuates HFD-induced obesity by increasing energy expenditure.^316,317^ Consistently, pharmacological inhibition of TBK1 enhances insulin sensitivity and reduces chronic inflammation caused by obesity.^316,318,319^ However, the potential bidirectional roles of TBK1 in regulating inflammation should not be ignored, as it is found to promote STING ubiquitination and degradation and in turn elevate NF-κB activity and inflammation.^320^ Nevertheless, the crosstalk between TBK1 in the STING pathway and inflammation status and insulin resistance merits further investigation.

Altogether, these results indicate that a positive energy balance and overnutrition lead to abnormal inflammation responses in peripheral tissues/organs such as adipose tissue and intestinal tract, and this, in turn, drives some of the systemic metabolic alterations associated with obesity like impaired insulin sensitivity and decreased thermogenesis. Targeting the key molecules/pathways mediating the abnormal inflammatory status may be crucial for the management of obesity-related inflammation and complications.

## Drug-related signaling molecules and pathways

### GLP-1

GLP-1 is released by intestinal L‐cells and also by a discrete population of neurons in the caudal medulla.^321^ As an incretin, the circulating level of GLP-1 elevates severalfold after a meal, which partially depends upon mechanical forces such as gastric distension.^322,323^ Gastric distension also activates nucleus tractus solitarius (NTS) neurons to release GLP‐1,^324^ which contributes to the negative energy balance of central GLP‐1.^325^ In addition, both peripheral GLP-1 secretion and central GLP‐1 cellular activity are regulated by classic satiety factors such as cholecystokinin (CCK) and leptin.^326,327^ GLP-1 works by activating GLP-1 receptors (GLP-1Rs), which can couple to Gαs, Gαq, Gαi, and Gαo.^323,328–330^ GLP-1Rs are widely expressed in the CNS, in peripheral organs (such as the pancreas), and in peripheral nerves such as vagal afferents.^328,331–333^ By stimulating GLP-1R, GLP-1 leads to an increase in intracellular Ca^2+^ and adenylate cyclase (AC), the activation of cAMP-dependent protein kinase (PKA) and Epac2, and the subsequent activation of multiple signal transduction pathways such as MAPK, PI3K, and BMP4, thus regulating the transcription of target genes.^334^ The activation of GLP‐1R has potent effects on the regulation of appetite, gastric motility, glucose, lipid metabolism, and even body thermogenesis (Fig. 5). These effects have made GLP-1R a viable target for diabetes mellites and obesity therapies,^328^ which we will discuss later.

The mechanism involved in GLP-1/GLP-R-mediated satiation is complicated, and there may be two substantially different modes of action between the central and peripheral regions. Within the CNS, activation of NTS GLP‐1 neurons leads to an attenuation of metabolic rate and a reduction in food consumption.^335–337^ Notably, ablation or inhibition of NTS GLP‐1 neurons increased refeeding after a fast and inhibited stress‐induced hypophagia.^338^ This phenomenon is considered to be mediated by a “local circuit”. Neuron-produced GLP‐1 is transported to the axon terminals of the producing cells and is stored in synaptic vesicles until it is eventually released into the synaptic cleft or extrasynaptically released into the brain parenchyma.^339^ Considering that GLP‐1-producing neurons are also projecting neurons with axons containing GLP‐1 vesicles in many distinct regions of the brain, it is speculated that the release and action of GLP‐1 within the CNS is similar to that of other neurotransmitters and modulators, which are locally restricted. From this point of view, GLP‐1 released from a specific neuron only acts at the site of its release, and it is entirely determined by the CNS area to which these neurons project.^340^ For instance, GLP‐1R is coexpressed with POMC neurons independent of AgRP/NPY expression. Electrophysiological measurements of murine brain slices revealed that GLP-1 can directly stimulate POMC/CART neurons via transient receptor potential channel 5, whereas it indirectly inhibits neurotransmission in neurons expressing NPY and AgRP via GLP‐1R-dependent activation of presynaptic GABAergic neurons.^341,342^ The involved intracellular signaling is proposed to be that GLP-1R activation increased PKA and MAPK activity and decreased the phosphorylation of AMPK in the NTS.^343^

In contrast, peripheral GLP-1 potentially works throughout the entire body by acting on, for example, vagal nerve endings embedded into the gut mucosa or is transported freely to most sites in the body accessible from the circulation.^344^ Currently, strong evidence suggests that the satiation effects of gut-derived GLP-1 are primarily mediated by vagal afferents, which relay the information to the hypothalamus and other forebrain regions by way of ascending second-order neurons.^334^ Peripheral administration of a GLP-1–albumin recombinant fusion protein, which is much larger and unable to cross the blood–brain barrier, activates neurons in the CNS coupled to feeding and inhibits food intake in mice,^345^ suggesting that peripheral GLP-1 activates central neurons regulating energy intake without direct interaction with GLP-1R in CNS. In rats, peripheral GLP-1-induced anorexia and neuronal activation of hypothalamic feeding circuits were both precluded by bilateral vagotomy or surgical transection of the brainstem-hypothalamic pathway.^346^ Likewise, selectively ablating nodose ganglionic neurons and the vagus nerve via systemic treatment with capsaicin completely blocks the anorectic effect of peripherally administered exendin-4 in mice.^347^ Collectively, these findings indicate that food reduction induced by peripheral GLP-1 is CNS-dependent. It is worth noting that the “brain circuits” mediating satiation induced by GLP-1 originating from either the CNS or periphery have only been described in rodents, but knowledge is limited, and it is not clear whether this circuit exists in humans.

GLP-1 also shows inhibitory effects on pentagastrin- and meal-stimulated gastric acid secretion and gastric emptying. GLP-1-induced gastrointestinal motility inhibition is mediated through GLP-1R at the level of myenteric neurons, followed by downstream signaling of nitrergic and cAMP-dependent mechanisms, resulting in the inhibition of vagal activity.^348–350^ Targeting GLP-1R signaling via exendin or vagal afferent denervation abolishes the inhibitory effect of centrally or peripherally administered GLP-1 on gastric emptying and acid secretion.^351^ In addition, intraperitoneal administration of an albumin-linked GLP-1R agonist that is unable to cross the blood–brain barrier can still activate neurons in the CNS that are coupled to gastrointestinal motility and lead to the inhibition of gastric emptying.^345^ Collectively, these experimental data indicate that the inhibitory effect of GLP-1 on gastric emptying and acid secretion is vagus-dependent and involves GLP-1Rs and/or on vagal afferent fibers that relay sensory information from the digestive tract to the brainstem.

GLP-1 stimulates glucose-dependent insulin secretion by binding to its specific receptor on pancreatic cells. GLP-1R stimulation leads to the activation of AC activity and the production of cAMP,^352^ which is the primary effector of GLP-1–induced insulin secretion. cAMP stimulates insulin secretion via two distinct mechanisms: PKA-dependent phosphorylation of downstream targets and PKA-independent activation of Epac2.^352^ In vivo, GLP-1R agonists improve glucose tolerance, enhance β-cell proliferation and neogenesis, and inhibit β-cell apoptosis in experimental rodent models of diabetes, leading to increased β-cell mass.^353–355^ Obese diabetic db/db mice develop ER stress, and GLP-1R agonists not only decrease the weights of mice but also reduce the levels of ER stress markers and improve β-cell function and survival during ER stress in a PKA-dependent manner.^356^ In addition to stimulating insulin secretion, GLP-1 also plays an important role in improving insulin sensitivity in insulin-targeting organs/tissues such as the liver and adipose tissue, partially through AMPK-related pathways.^357–359^ This can be mediated by its direct actions on peripheral tissue by improving glyoxalase activity^359^ and via CNS signals, which is suggested by the evidence that central GLP-1R antagonism attenuated the remission in HFD-induced insulin resistance caused by peripheral GLP-1 infusion.^360^ Although some evidence indicates that GLP-1R in the brain is not necessary for physiologic control of glucose regulation, the central actions of GLP-1R signaling should not be ignored given its critical role in lowering weight, which is the primary goal for T2D and also other metabolic disorders.

GLP-1 signaling is also a regulator of adipogenesis. Growing in vitro evidence revealed that GLP-1R activation increased the expression of differentiation marker genes such as PPARγ and FABP4 and lipid accumulation during preadipocyte differentiation.^361^ Gut-derived GLP-1 also increases adipocyte mass through preadipocyte proliferation and inhibition of apoptosis,^362^ which is partially mediated by the PI3K, MAPK, and Wnt4-β-catenin pathways.^362–364^ Notably, although GLP-1 signaling seems to promote preadipocyte differentiation both in vivo and in vitro, it decreased fatty acid synthase expression in mature adipocytes,^361^ an enzyme closely related to lipogenesis and the development of visceral obesity.^365–367^ Considering that adipocyte enlargement plays the leading role during lipogenesis and obesity, while adipocyte differentiation can offset the negative metabolic effects of obesity,^291,368^ the terminal effect of GLP-1 on metabolism may still be positive.

GLP-1R activation was found to directly increase lipolysis and fatty acid oxidation by upregulating Sirt1 expression in differentiated 3T3-L1 adipocytes.^369^ It also enhances lipolysis by promoting BAT thermogenesis or white adipocyte browning.^370,371^ Recent studies revealed that GLP-1R located in the epicardial adipose tissue (EAT) was directly correlated with genes promoting beta-oxidation and white-to-brown adipocyte differentiation but inversely correlated with pro-adipogenic genes,^372^ while EAT is a risk factor for cardiovascular diseases,^373^ suggesting that GLP-1R may be a new target to modulate cardiovascular risk related to obesity. GLP-1 signaling participates in the process of thermogenesis in BAT by inhibiting the BMP4-related signaling pathway in HFD-induced obese mice.^370^ Central signaling may play a role in GLP-1-induced thermogenesis since GLP-1 administered into the dorsomedial hypothalamus of rats increases BAT thermogenesis and triglyceride mobilization in the liver, whereas loss of GLP-1 signaling in the dorsomedial hypothalamus area reduces BAT thermogenesis and increases adiposity.^374^ Similar results were observed in a mouse model; central injection of a clinically used GLP-1R agonist, liraglutide, stimulates BAT thermogenesis and white adipocyte browning independent of nutrient intake.^225^ Activation of AMPK in the hypothalamic ventromedial nucleus (VMN) blunted both central liraglutide-induced thermogenesis and adipocyte browning.^225^ These data indicate that GLP-1 lowers body weight by regulating either food intake or energy expenditure through various hypothalamic sites and that these mechanisms might be clinically relevant.

### Melanocortin signaling pathway

**Fig. 6:**
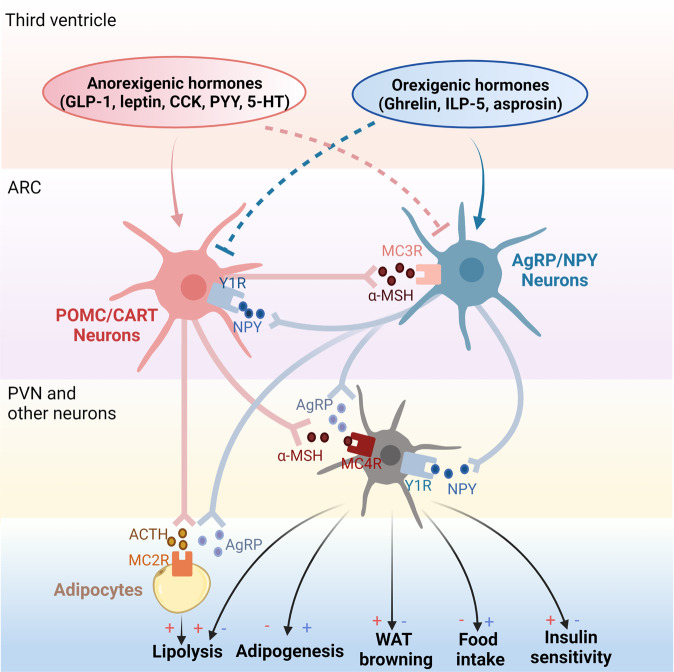
Melanocortin pathway in obesity pathogenesis. The melanocortin pathway consists of POMC; melanocortin receptors MC1R-MC5R; and agouti and AgRP. POMC/CART neurons in ARC are stimulated by anorexigenic hormones in the third ventricle like GLP-1, leptin, CCK, PYY, and 5-HT, while suppressed by orexigenic hormones like Ghrelin, ILP-5, and asprosin. Upon stimulation, POMC/CART neurons secrete POMC including α-MSH and ACTH. α-MSH is released into the PVN. By interacting with MC4R, α-MSH activates PVN neurons and displays anti-obesity effects by inhibiting adipogenesis, promoting lipolysis, inducing WAT browning, reducing food intake, and improving insulin sensitivity. ACTH released by POMC/CART neurons actions on adipocytes directly by binding to MC2R, further promoting lipolysis. However, these effects can be abolished by AgRP, which is the endogenous antagonist of POMC and is secreted by AgRP/NPY neurons in ARC. Conversely, AgRP/NPY neurons can be stimulated by orexigenic hormones in the third ventricle but inhibited by anorexigenic hormones. Notably, POMC/CART and AgRP/NPY neurons interact mutually. NPY receptor Y1R is expressed in POMC/CART neurons and its activation inhibits POMC neurons in the ARC. In contrast, MC3R expressed in AgRP/NPY neurons seems to increase food intake in an “AgRP circuitry”-dependent manner

The melanocortin signaling pathway consists of a set of hormonal and neuropeptidergic networks with three major components: pro-peptide POMC, which is posttranslationally processed by prohormone convertases into a number of biologically active moieties, including α-MSH, β-MSH, γ-MSH, and adrenocorticotrophin (ACTH);^375^ the five G protein-coupled melanocortin receptors, MC1R-MC5R, that mediate their actions;^376^ and endogenous antagonists of those receptors, agouti, and AgRP.^377,378^ Although its mechanism of action is not yet clear, it is certain that the melanocortin signaling pathway plays a key role in the development of obesity by regulating energy homeostasis (Fig. 6), and compounds targeting the melanocortin system have been investigated extensively from basic to clinical research for anti-obesity purpose.

The melanocortin system serves as a major regulator of food intake and energy balance.^378,379^ ARC POMC neurons are activated by multiple anorexigenic hormones including leptin, GLP-1, CCK, peptide tyrosine-tyrosine (PYY), etc., but inhibited by orexigenic hormones, such as ghrelin, asprosin, and insulin-like peptide 5 (ILP-5).^380–384^ In general, POMC neurons are more active in post nutritional repletion states than in fasting states,^385^ indicating negative feedback exists in the regulation of food intake. Endocrine factors that activate POMC neurons cause anorexia and weight loss.^384^ These effects are mimicked by local application of α-MSH and are diminished in mice that lack MC4R,^386^ indicating that α-MSH signaling through the MC4R axis is at least partly responsible for some of these endocrine effects. Over the past 20 years, the role of ARC POMC neurons in response to peripheral factors, particularly the adipocyte-derived hormone leptin, has been extensively studied. Leptin is a hormone secreted by adipocytes to modulate several neuroendocrine functions.^387^ LEPRs are widely expressed in the CNS, particularly in some regions of the hypothalamus regulating feeding.^388^ By activating LEPR in ARC, leptin modulates the activity of the melanocortin pathway by enhancing the α-MSH cleaved from POMC but blunting the synthesis of NPY and AgRP, to activate MC4R axis and thus exhibits an anorectic effect.^389,390^ Population studies have identified that the deficiency of LEPR caused by the mutations in LEPR gene leads to severe obesity.^391^ Despite several variants of MC4R being associated with significantly lower BMI and lower odds of obesity, most MC4R variants cause loss of function and increase obesity risk.^392,393^ In contrast, activation of AgRP neurons leads to hyperphagia and weight gain. Notably, a recent study revealed that the chronic activation of AgRP GABA+ neurons or non-AgRP GABA+ neurons both leads to obesity, while inhibition of arcuate GABA+, but not AgRP neurons reduces weight gain, indicating that arcuate GABA+ neurons may be the major mediator to increase food intake.^378^ In addition, appetite is suppressed in mice lacking MC3R.^394^ Considering that MC3R is expressed in 97% of AgRP/NPY neurons and pharmacological effects of MC3R compounds on feeding are dependent on intact AgRP circuitry in mice,^395^ the dominant effect of MC3R appears to be the regulation of the AgRP circuitry. Notably, the NPY receptor Y1R is expressed in POMC/CART neurons,^396^ and its activation inhibits POMC neurons through the Y1R-mediated activation of G protein-gated inwardly rectifying potassium channel currents.^397^ These results indicate that there is an interplay between these two peptides at multiple levels.^70^

It has been recently proposed that leptin and insulin also act on POMC neurons to increase energy expenditure via a pathway that involves protein-tyrosine phosphatases 1B and T-cell protein-tyrosine phosphatase and leads to increased browning differentiation of WAT.^158,398^ Regarding lipid metabolism, Lede et al. analyzed transcriptome changes and found significant alterations in components of triacylglycerol metabolism, unsaturated fatty acid biosynthesis, PPAR signaling pathways, and lipid transport and storage in MC4R-deficient mice compared to the wild-type condition.^399^ Furthermore, Iqbal et al. found that LEPR deficiency resulted in lipid accumulation in the intestine, liver, and plasma.^400^ The molecular mechanism was decreased intestinal microsomal triglyceride transfer protein expression, reduced assembly and secretion of lipoproteins, and elevated triglyceride accumulation.^400^

The melanocortin system also contributes to the regulation of glucose metabolism. Mouse models and humans with genetic deficiency of POMC or MC4R show significant hyperinsulinemia and insulin resistance.^401–403^ Conversely, activation of brain MC4R enhances insulin sensitivity.^404^ Similarly, the direct leptin action on POMC neurons lowers glucagon levels and improves hepatic insulin sensitivity.^405^ Moreover, nutritional status modulates insulin responsiveness in POMC neurons.^406^ Another population of hypothalamic POMC neurons that regulates both energy and glucose homeostasis has been found to express the serotonin (5-hydroxytryptamine [5-HT]) receptor 2C receptor, which signals to induce activation of TrpC5 and the mTOR pathway.^407–410^ A recent study suggested that the 5’−3’ exoribonuclease XRN1 inhibits AgRP neuron function.^411^ Together, these studies highlight an important role of the melanocortin pathway in the regulation of obesity and glucose homeostasis.

Melanocortins circulate throughout the body and exert lipolytic effects on adipocytes via specific melanocortin receptor subtypes.^412^ Obesity, which is caused by overexpression of AgRP, is generally considered a consequence of antagonism of α-MSH on the hypothalamic melanocortin receptor, given that AgRP stimulates adipogenesis and antagonizes melanocortin-mediated lipolysis in adipocytes.^413^ Moreover, PPARγ, a critical transcription factor in the regulation of adipocyte differentiation and lipid metabolism,^414–416^ was reported to regulate transcriptional activation of the MC2R accessory protein gene to stimulate lipolysis induced by ACTH in mature adipocytes.^417^ Similarly, Mynatt et al. utilized engineered transgenic mice with agouti overexpression in adipose tissue as well as differentiated 3T3-L1 adipocytes and observed an elevation of PPARγ expression in both models, suggesting that PPARγ, probably interacting with ACTH and AgRP, to regulate adipocyte differentiation.^418^

## Drugs/compounds for the treatment of obesity

**Fig. 7:**
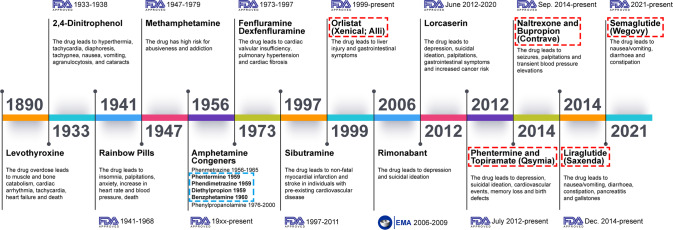
Timeline of anti-obesity medications approved by the FDA or EMA from the late nineteenth century until today (the red dashed line indicates long-term use while the blue dashed line indicates short-term use)

Lifestyle interventions remain the cornerstone of weight management, but most patients cannot achieve long-term meaningful weight loss simply by changing lifestyles. Thus, pharmacotherapy is appropriate after lifestyle modification failure and is recommended as an adjunct to individuals with BMI ≥30 kg/m^2^ or BMI ≥27 kg/m^2^ with obesity-associated comorbidities.^419^ Currently, the U.S. Food and Drug Administration (FDA) has approved four AOMs that curb appetite (phentermine, phendimetrazine, diethylpropion, and benzphetamine) for short-term (≤12 weeks) use and five AOMs (orlistat, phentermine-topiramate, naltrexone-bupropion, liraglutide, and semaglutide) for long-term use and another drug setmelanotide for people with obesity due to three specific rare genetic conditions (Fig. 7). There has been a long-term effort to develop new weight-loss drugs, while most results have been disappointing, several prominent classes of targets have caught the attention of the scientific community and drugmakers (Table 1).

**Table 1:** Weight-loss drugs in clinical development after 2015

Agent type	Agent	Indication	Manufacturer	Trials	ClinicalTrials.gov ID/ref.
*Energy intake*					
MC4R agonist	PL-8905	Obesity	Palatin Technologies	Announced	Not Recorded
NPY5R antagonist	S-237648	Obesity	Shionogi & Co.	In-house	See R&D Pipeline
Triple reuptake inhibitor/SNDRI	Tesofensine/NS-2330	Obesity	NeuroSearch A/S	Phase 2	NCT00394667
Peripheral CB1 receptor blocker	GFB-024 (inverse agonist)	Kidney diseases	Goldfinch Bio	Phase 1	NCT04880291
	AM-6545 (antagonist)	Obesity	MAKScientific	Preclinical	See R&D Pipeline
GLP-1R agonist	Beinaglutide/Benaglutide	Obesity	Shanghai Benemae	Phase 3	NCT03986008
	Dulaglutide	T2D	Eli Lilly and Company	Phase 3	NCT03015220
				Phase 2	NCT02973100
				Phase 4	NCT02750410
	LY3502970	Obesity, T2D	Eli Lilly and Company	Phase 1	NCT05086445
				Phase 2	NCT05051579
				Phase 2	NCT05048719
	Efpeglenatide/^LAPS^Exd4 Analog	T2D	Hanmi Pharmaceutical	Phase 3	NCT03353350
	Exenatide	HO	AstraZeneca	Phase 3	NCT02860923
	PB-119	T2D	PegBio Co.	Phase 3	NCT04504396
				Phase 3	NCT04504370
	Danuglipron/PF-06882961	Obesity, T2D	Pfizer	Phase 2	NCT04707313
				Phase 2	NCT04617275
				Phase 2	NCT03985293
	PF-07081532	Obesity, T2D	Pfizer	Phase 1	NCT04305587
	RGT001-075	T2D	Regor Therapeutics	Phase 2	NCT05297045
	Noiiglutide/SHR20004	Obesity	Hansoh Pharma	Phase 2	NCT04799327
	TG103	Obesity	CSPC Pharmaceutical	Phase 2	NCT05299697
	TTP273	T2D	vTv Therapeutics	Phase 2	NCT02653599
	XW003	Obesity	Sciwind Biosciences	Phase 2	NCT05111912
	XW004	Obesity, T2D	Sciwind Biosciences	Phase 1	NCT05184322
GCGR agonist	HM15136/^LAPS^Glucagon Analog	Obesity, T2D	Hanmi Pharmaceutical	Phase 1	NCT04167553
	NN9030/NNC9204-0530	Obesity	Novo Nordisk	Phase 1	NCT02835235
GIPR agonist	ZP 6590	Obesity	Zealand Pharma	Preclinical	See R&D Pipeline
GLP-1R/GCGR dual agonist	Pemvidutide/ALT-801	Obesity	Altimmune	Phase 2	NCT05295875
	BI 456906	Obesity	Boehringer Ingelheim	Phase 2	NCT04667377
	CT-388	T2D	Carmot Therapeutics	Phase 1	NCT04838405
	CT-868	Obesity, T2D	Carmot Therapeutics	Phase 2	NCT05110846
	DD01	Obesity, T2D	D&D Pharmatech	Phase 1	NCT04812262
	JNJ-64565111	Obesity, T2D	Johnson & Johnson	Phase 2	NCT03586830
				Phase 2	NCT03486392
	NN9277/NNC9204-1177	Obesity	Novo Nordisk	Phase 1	NCT02941042
	Efinopegdutide/^LAPS^GLP/GCG	NASH	Hanmi Pharmaceutical	Phase 2	NCT03486392
	SAR425899	T2D	Sanofi	Phase 1	NCT03376802
	OXM analog—Cotadutide/MEDI0382	T2D	AstraZeneca	Phase 1	NCT02548585
				Phase 1	NCT04208620
	OXM analog—G3215	Obesity, T2D	Imperial College London	Phase 1	NCT02692040
	OXM analog—IBI362/LY3305677	Obesity, T2D	Eli Lilly and Company	Phase 2	NCT04904913
				Phase 1	NCT04440345
	OXM analog—MOD-6031	Obesity, T2D	OPKO Health	Phase 1	NCT02692781
	OXM analog—OPK-88003/LY2944876	Obesity, T2D	OPKO Health	Phase 2	NCT03406377
GLP-1R/GIPR dual agonist	HS-20094	T2D	Hansoh Pharma	Phase 1	NCT05116410
	Tirzepatide/LY3298176	Obesity, T2D	Eli Lilly and Company	Phase 3	NCT05024032
				Phase 3	NCT04844918
				Phase 3	NCT04657016
				Phase 3	NCT04660643
				Phase 3	NCT04657003
				Phase 3	NCT04184622
GLP-1R/GIPR/GCGR triple agonist	HM15211/^LAPS^Triple Agonist	NASH	Hanmi Pharmaceutical	Phase 2	NCT04505436
	LY3437943	Obesity, T2D	Eli Lilly and Company	Phase 1	NCT04823208
				Phase 2	NCT04881760
				Phase 2	NCT04867785
	NN9423/NNC9204-1706	Obesity	Novo Nordisk	Phase 1	NCT03095807
	NNC0480-0389				
	SAR441255	Obesity	Sanofi	Phase 1	NCT04521738
GLP-1R agonist and GIPR antagonist	AMG133	Obesity	Amgen	Phase 1	NCT04478708
	GMA106	Obesity	Gmax Biopharm	Phase 1	NCT05054530
DPP-4 inhibitor	HSK7653	T2D	Haisco Pharmaceutical	Phase 3	NCT04556851
	Sitagliptin	T2D, NAFLD	Merck & Co.	Phase 4	NCT05195944
				Phase 3	NCT02849080
	Yogliptin	Obesity, T2D	Easton Biopharmaceuticals	Phase 3	NCT05318326
AMYR agonist	Cagrilintide/NN9838/AM833/NNC0174-0833	Obesity, T2D	Novo Nordisk	Phase 1	NCT04940078
				Phase 1	NCT05254158
				Phase 2	NCT03856047
	ZP8396	Obesity	Zealand Pharma	Phase 1	NCT05096598
AMYR/CTR dual agonist	KBP-042	T2D	Nordic Bioscience	Phase 2	NCT03230786
	KBP-089	T2D	Nordic Bioscience	Phase 1	NCT03907202
TAS2R agonist	ARD-101	Obesity	Aardvark Therapeutics	Phase 2	NCT05121441
PYY/Y2R signaling	NNC0165-1562	Obesity	Novo Nordisk	Phase 1	NCT03574584
	PYY1875/NNC0165-1875	Obesity	Novo Nordisk	Phase 1	NCT03707990
	NN9748/NN9747	Obesity, T2D	Novo Nordisk	Phase 1	NCT03574584
Ghrelin signaling	NOX-B11	Obesity	NOXXON Pharma	Preclinical	Not Recorded
	GLWL-01	PWS	GLWL Research	Phase 2	NCT03274856
	RM-853/T-3525770	PWS	Rhythm Pharmaceuticals	Preclinical	See R&D Pipeline
	TZP-301	Obesity	Ocera Therapeutics	Preclinical	Not Recorded
	EX-1350	Obesity, T2D	Elixir Pharmaceuticals	Preclinical	Not Recorded
Leptin analog	Metreleptin	Lipodystrophy	AstraZeneca	Phase 3	NCT05164341
Leptin sensitizer	Celastrol	Obesity, T2D	Research Use Only	Preclinical	PMID: 26000480^420^
	Withaferin A	Obesity, T2D	Research Use Only	Phase 1	PMID: 30904387^421^
	ERX1000	Obesity	ERX Pharmaceuticals	Phase 1	NCT04890873
GDF15 agonist	LA-GDF15	Obesity	Novo Nordisk	Phase 1	See R&D Pipeline
	LY3463251	Obesity	Eli Lilly and Company	Phase 1	NCT03764774
α7-nAChR agonist	GTS-21/DMXB-A	Obesity	Otsuka Pharmaceutical	Phase 1	NCT02458313
*Energy absorption*					
Strain product	WST01	Obesity, T2D	SJTUSM	Phase 2	NCT04797442
	Xla1	Obesity	YSOPIA Bioscience	Phase 1	NCT04663139
Orlistat and acarbose	EMP16-02	Obesity	Empros Pharma AB	Phase 1	NCT04521751
*Energy storage*					
MGAT2 inhibitor	BMS-963272	Obesity	Bristol Myers Squibb	Phase 1	NCT04116632
	S-309309	Obesity	Shionogi & Co.	In-house	See R&D Pipeline
DGAT2 inhibitor	Ervogastat/PF-06865571	NASH, NAFLD	Pfizer	Phase 1	NCT03513588
Sirt1/AMPK/eNOS signaling	NS-0200/Leucine-Metformin-Sildenafil	Obesity	NuSirt Biopharma	Phase 2	NCT03364335
Labisia pumila extract	SKF7	Obesity	Medika Natura	Phase 2	NCT04557267
Stimulating IDE synthesis	Cyclo-Z (cyclo(his-pro) plus zinc)	T2D	NovMetaPharma	Phase 2	NCT03560271
					NCT02784275
αGI inhibitor	Sugardown/BTI320	Prediabetes	Boston Therapeutics	Phase 2	NCT02358668
CCR2/CCR5 dual agonist	Cenicriviroc	T2D, NAFLD	AbbVie	Phase 2	NCT02330549
*Energy expenditure*					
SGLT2 inhibitor	Ipragliflozin/ASP1941	T2D	Astellas Pharma	Phase 3	NCT02452632
	Bexagliflozin/EGT1442	T2D	Theracos	Phase 3	NCT02836873
				Phase 3	NCT02715258
				Phase 3	NCT02558296
	Remogliflozin etabonate	T2D	Avolynt	Phase 2	NCT02537470
	Canagliflozin	Obesity, T2D	Johnson & Johnson	Phase 4	NCT02360774
	Dapagliflozin	T2D, HF, CKD	AstraZeneca	Phase 2	NCT05179668
				Phase 4	NCT04249778
				Phase 2	NCT03968224
				Phase 3	NCT02413398
	Empagliflozin	T1D, T2D	Boehringer Ingelheim	Phase 3	NCT04233801
				Phase 2	NCT03132181
				Phase 4	NCT03157414
				Phase 3	NCT02863328
				Phase 3	NCT02580591
				Phase 3	NCT02414958
	Ertugliflozin	T2D, HF	Merck & Co.	Phase 3	NCT03717194
SGLT1/2 inhibitor	Licogliflozin/LIK066	Obesity	Novartis	Phase 2	NCT03320941
				Phase 2	NCT03100058
	Sotagliflozin	T1D, T2D, CKD	Lexicon Pharmaceuticals	Phase 3	NCT03242252
				Phase 3	NCT03242018
				Phase 3	NCT02531035
				Phase 3	NCT02384941
MetAP2 inhibitor	Beloranib/ZGN-440/ZGN-433	Obesity	Larimar Therapeutics	Phase 2	NCT01666691
	ZGN-1061	Obesity, T2D	Larimar Therapeutics	Phase 2	NCT03254368
FGF21/FGFR1c/β-Klotho signaling	LLF580	Obesity	Novartis Pharmaceuticals	Phase 1	NCT03466203
	NN9499/NNC0194-0499	Obesity	Novo Nordisk	Phase 1	NCT03479892
	MK-3655/NGM313	Obesity, NASH	Merck & Co.	Phase 1	NCT02708576
					NCT04583423
	BFKB8488A	NAFLD	Genentech	Phase 1	NCT02593331
FGFR4 inhibitor	IONIS-FGFR4Rx	Obesity	Ionis Pharmaceuticals	Phase 2	NCT02476019
FXR agonist	ASC42	Obesity, NASH	Gannex Pharma	Phase 1	See R&D Pipeline
THR-β agonist	ASC41	Obesity, NAFLD	Gannex Pharma	Phase 1	NCT04686994
sGC stimulator	Praliciguat/IW-1973	T2D	Cyclerion Therapeutics	Phase 2	NCT02906579
Neutrophil elastase inhibitor	PHP-303	Obesity	pH Pharma	Phase 1	NCT03775278
PDE4/5 inhibitor	Roflumilast	Obesity	Altana Pharma	Phase 3	NCT04800172
	Tadalafil	Obesity	Eli Lilly and Company	Phase 2	NCT02819440
Glabridin analog	HSG4112	Obesity	Glaceum	Phase 1	NCT05310032
				Phase 2	NCT05197556
				Phase 1	NCT04703764
ActRII inhibition	Bimagrumab/BYM338	T2D	Novartis	Phase 2	NCT03005288

*MC4R* melanocortin-4 receptor, *NPY5R* neuropeptide y receptor y5, *R&D* research and development, *SNDRI* serotonin–norepinephrine–dopamine reuptake inhibitor, *CB1* cannabinoid receptor 1, *GLP-1R* glucagon-like peptide 1 receptor, *T2D* type 2 diabetes, *LAPS* long-acting peptide/protein discovery, *HO* hypothalamic obesity, *GCGR* glucagon receptor, *GIPR* glucose-dependent insulinotropic polypeptide/gastric inhibitory polypeptide receptor, *NASH* nonalcoholic steatohepatitis, *OXM* oxyntomodulin, *DPP-4* dipeptidyl peptidase-4, *NAFLD* nonalcoholic fatty liver disease, *AMYR* amylin receptor, *CTR* calcitonin receptor, *TAS2R* bitter taste receptor class 2, *PYY* peptide tyrosine-tyrosine, *Y2R* neuropeptide y receptor type 2, *PWS* Prader–Willi syndrome, *GDF15* growth differentiation factor 15, *α7-nAChR* alpha7 nicotinic acetylcholine receptor, *MGAT2* monoacylglycerol acyltransferase 2, *DGAT2* diacylglycerol acyltransferase 2, *IDE* insulin degrading enzyme, *αGI* alpha-glucosidase inhibitor, *CCR2/5* C-C chemokine receptor type 2/5, *SGLT1/2* sodium-glucose cotransporter 1/2, *HF* heart failure, *CKD* chronic kidney disease, *T1D* type 1 diabetes, *MetAP2* methionine aminopeptidase 2, *FGF21* fibroblast growth factor 21, *FGFR1c* fibroblast growth factor receptor 1c isoform, *FGFR4* fibroblast growth factor receptor 4, *FXR* farnesoid x receptor, *THR-β* thyroid hormone receptor beta, *sGC* soluble guanylate cyclase, *PDE4/5* phosphodiesterase-4/5, *ActRII* activin type II receptors

Zealand Pharma R&D Pipeline: https://www.zealandpharma.com/product-pipeline

Rhythm Pharmaceuticals R&D Pipeline: https://www.rhythmtx.com/our-pipeline-old

Shionogi & Co. R&D Pipeline: https://www.shionogi.com/global/en/innovation/pipeline.html

MAKScientific R&D Pipeline: https://makscientific.com/drug_pipeline.html

Gannex Pharma R&D Pipeline: https://www.gannexpharma.com/portal/list/index/id/9.html

Novo Nordisk R&D Pipeline: https://www.novonordisk.com/science-and-technology/r-d-pipeline.html

### The chronology of AOMs

The hunt for AOMs dates back to the late nineteenth century (Fig. 7). A 50-year-old female who was about 160 cm tall and weighed 112 kg died of levothyroxine abuse because of her obsession with losing weight.^422^ Soon after, there was another case report of using sheep-derived thyroid extract to increase metabolic rate for weight loss.^423^ The 2,4-dinitrophenol was all the rage for its impressive weight-lowering effect in the 1930s, but it came with lethal side effects and was suspended by the FDA in 1938 (Fig. 7).^424–427^ Undiscouraged by these failures, the pharmaceutical companies in the weight loss industry have been trying to seek for a panacea to beat obesity. In 1941, the Clark & Clark (Camden, NJ) combined amphetamine, thyroid, and drugs that targeted untoward effects, and named it Clarkotabs, or the rainbow pills, creating the first commercial diet pills.^427,428^ The rainbow pills enjoyed a high reputation until 1968 when the FDA prohibited their manufacture and marketing due to findings from the U.S. Senate that the rainbow pills killed over 60 persons.^428^ Methamphetamine and amphetamine congeners (phenmetrazine, phendimetrazine, phentermine, diethylpropion, benzphetamine, cathine, phenylpropanolamine) were well received given their anorectic effect and were approved by the FDA from 1947 to 1976 to manage obesity.^427,429–434^ Fenfluramine, a serotonergic agent, was approved by the FDA to lower body weight in 1973. The drug was later coupled with phentermine (fen-phen), resulting in an anorectic that exhibited a balanced norepinephrine-serotonin (5-HT) release.^426,427,432,433,435,436^ Despite fen-phen having never been approved by the FDA, the number of Americans who were prescribed fen-phen exceeded 18 million in 1996.^427,437,438^ In the same year, the FDA considered dexfenfluramine safe for use and gave a seal of approval.^427,429,433^ However, fenfluramine and dexfenfluramine were reported to be closely related to valvular heart disease, pulmonary hypertension, and cardiac fibrosis, and were withdrawn from distribution by the FDA in 1997.^427,433,435,438–442^ That same year, the FDA approved sibutramine for the long-term treatment of obesity and banned the sale of sibutramine in 2011 over concerns about myocardial infarction and stroke.^433,443–446^ Although the FDA ceased the commercialization of fenfluramine in 1997, phentermine, once used in combination with fenfluramine as fen-phen, is still approved to treat obesity. In July 2012, the FDA approved phentermine/topiramate extended-release (Qsymia) as an adjunct to lifestyle interventions for long-term weight management.^447–450^ Orlistat (Xenical; Alli), approved for weight loss by the FDA in 1999, inhibits gastrointestinal lipase and thereby blocks the absorption of dietary fat by about 30%.^451–457^ Cannabinoid receptor type 1 (CB1) is one of the major receptors of the endocannabinoid system and is widespread in the CNS, including regions that control food intake. Rimonabant is a highly selective CB1 receptor blocker that antagonizes CB1 through inverse agonism, thereby modulating neurocircuits controlling homeostatic feeding and hedonic feeding to shed unwanted pounds.^458–464^ In 2006, the rimonabant is fully accredited by the European Medicines Agency for use in the European Union.^465^ However, rimonabant antagonized CB1 in the ventral tegmental area (VTA) and amygdala leading to depression and suicidal ideation, prompting its abolition in 2009.^466–468^ Lorcaserin is a highly selective 5-HT_2C_ receptor agonist, while its affinity for other 5-HT receptors is greatly reduced. In light of the success of lorcaserin in weight-loss trials, the FDA approved it for long-term weight control in June 2012.^469–474^ In 2020, the FDA called for the discontinuation of lorcaserin as clinical trials had shown increased cancer rates.^475^ In September and December 2014, naltrexone extended-release/bupropion extended-release (Contrave) and liraglutide (Saxenda) were approved by the FDA for obesity treatment in succession.^449,476–486^ Semaglutide (Wegovy) is the second FDA-approved GLP-1R agonist targeting obesity after liraglutide.^487–495^ Compared with an average of about 5–10% of body weight loss achieved with other currently FDA-approved drugs, semaglutide reaches an approximately 15% average weight loss, ushering in a new era against obesity.^496^ In November 27, 2020, the FDA approved a melanocortin-4 receptor (MC4R) agonist setmelanotide (IMCIVREE) for chronic weight management in the obese aged ≥6 years in the setting of proopiomelanocortin, proprotein convertase subtilisin/kexin type 1, and LEPR deficiency.^497,498^

### Currently approved AOMs

Phendimetrazine, phentermine, diethylpropion, and benzphetamine are amphetamine congeners functionally identical to amphetamine, which can curb appetite and act as FDA-approved sympathomimetic medications for weight management, but only for short-term use due to safety concerns.^499^ Amphetamine congeners are competitive substrates for the norepinephrine (NE) transporter (NET), dopamine (DA) transporter (DAT), and 5-HT transporter (SERT).^500,501^ They bind to NET and DAT with 500-fold greater affinity compared with SERT, and therefore harness monoamines as neurotransmitters mostly in catecholamine neurons in the reward and executive function pathways of the brain to exert its behavioral effects.^500–504^ NE and DA are the prime monoamine neurotransmitters and their concentrations in the synaptic cleft are increased by amphetamine congeners in a dose-dependent manner.^503,504^ Upon entering the presynaptic neuron, the amphetamine congeners encounter vesicular monoamine transporter type 2. Their interaction collapses the vesicular pH gradient and jeopardizes the acidic environment of the vesicle, preventing the translocation of NE and DA from the axoplasm into vesicles, and causing intracellular accumulation of NE and DA.^505,506^ Alternatively, amphetamine congeners target monoamine oxidase (MAO) and hinder MAO-mediated NE and DA breakdown, resulting in elevated intracellular concentrations of NE and DA. In fact, the distribution of the two isoenzymes of MAO (MAO-A and MAO-B) varies. The former is located at the synaptic terminals of NE and DA neurons, while the latter is the only isoform that acts at 5-HT terminals and within non-catecholamine neurons. Thus, the effect of amphetamine congeners on the number of extracellular monoamines is significant with regard to NE and DA, but less so for 5-HT.^500^ Amphetamine congeners further bind to the trace amine-associated receptor 1 and activate PKA and protein kinase C, triggering DA efflux and DAT internalization.^501,503^ Besides, amphetamine congeners also increase intracellular calcium, leading to DAT phosphorylation and subsequent DA efflux.^507,508^ All these amphetamine congeners-mediated processes contribute to NE and DA release in reward circuitry and executive functioning via NET and DAT, respectively. Elevated NE and DA produces a sense of satiety by activating postsynaptic NE and DA receptors.^501^

Qsymia is a combination of phentermine, a sympathomimetic amine anorectic, and topiramate extended-release, an antiepileptic drug. The pharmacologic activity of phentermine is akin to that of its prototype drug, amphetamine, whose mechanism of action has been elucidated in the above paragraph. However, the mechanism by which topiramate is able to manage weight in the long term remains uncharted and requires more in-depth investigations. Topiramate barricades voltage-gated sodium (Na^+^) and high voltage-activated calcium (Ca^+^) channels and positively modulates at least one potassium (K^+^) channel in presynaptic excitatory neurons. Topiramate has inhibitory effects on the α-amino-3-hydroxy-5-methyl-4-isoxazolepropionic acid (AMPA) and kainate receptors on postsynaptic neurons, both of which are ionotropic transmembrane receptors for glutamate. Topiramate enhances GABA synthesis and blocks its reuptake or degradation, which augments the activity of GABAergic neurons and positively modulates GABAA receptors. Besides, topiramate inhibits carbonic anhydrase isoenzymes.^509–515^

Contrave is the trade name of the anti-obesity combination of naltrexone hydrochloride 8 mg and bupropion hydrochloride 90 mg. Bupropion is a weak inhibitor of NE and DA reuptake, generally prescribed for anti-depression and smoking cessation aid.^516,517^ Naltrexone is a high-affinity antagonist for mu(μ)-opioid receptor (MOPr), primarily prescribed for the management of alcoholism and opioid addiction.^516,517^ Since bupropion and naltrexone reduce weight in the course of treatment, they were combined as a diet drug.^518^ Food intake is regulated by the melanocortin and NPY systems in the ARC of the hypothalamus.^519^ The melanocortin system contains POMC and AgRP cell populations, and adjusts homeostatic energy balance. POMC cells release α-MSH and β-endorphin. α-MSH activates MC4R, which in turn produces anorexic effects that increase energy expenditure and decrease appetite. The attachment of β-endorphin to the inhibitory MOPr on POMC cells spoils the activity of POMC cells. In contrast, AgRP and NPY are orexigenic peptides with appetite-stimulating effects, and ablation and stimulation of AgRP/NPY peptides can lead to decreased and increased food intake, respectively.^520^ AgRP is an MC4R antagonist that competitively blocks the α-MSH-MC4R cascade, reducing energy expenditure and increasing appetite. Bupropion inhibits the reuptake of NE and DA, provoking POMC neurons in the ARC of the hypothalamus. By releasing both α-MSH and β-endorphin, the β-endorphin-mediated autoinhibitory feedback effect neutralizes the positive signal of weight loss generated by α-MSH, which may interpret the limited long-term weight loss (<5%) of bupropion monotherapy. Intriguingly, naltrexone is able to antagonize MOPr and block its binding with β-endorphin, thereby preventing feedback autoinhibition of POMC neurons. Therefore, the naltrexone/bupropion combination exhibits stronger stimulation of POMC cells than either drug alone. Enhanced POMC signaling underlies the clinically effective weight loss of Contrave.^516–518,521^

Liraglutide (Saxenda) and semaglutide (Wegovy) are GLP-1R agonists, which help shed pounds via food intake reduction in that they lower appetite and inhibit gastric emptying.^322,522^ GLP‐1 mainly originates from the intestinal enteroendocrine L cells and the preproglucagon neurons (named after the transcript) or glucagon neurons (named after the gene) located in the hindbrain NTS.^523,524^ Conventional or habitual thinking holds that the peripheral (gut‐derived and exogenous) and central (brain‐derived) GLP-1 systems are connected, but current shreds of evidence suggest that they are most likely independent entities.^524–526^ In other words, compared with brain‐derived GLP‐1 released from the preproglucagon neurons, gut‐derived GLP‐1 released from the enteroendocrine L cells and exogenous GLP‐1 act in different modes of action to inhibit feeding behavior. Brierley et al. corroborated this point finding that exogenous liraglutide and semaglutide displayed intact ability for losing weight in the setting of ablative preproglucagon neurons.^325^ It appears that liraglutide and semaglutide, administered systemically, mimic the function of postprandial gut-derived GLP-1 and directly interact with GLP-1 receptors in the CNS that are not shielded by the blood–brain barrier to exert their slimming effect.^524,525^ First, liraglutide and semaglutide activate NTS GLP-1R signaling primarily by coupling to Gαs/Gsα, which simultaneously activates AMPK and suppresses ERK1/2 signaling pathways via increasing PKA activity, thereby increasing cAMP response element-binding protein (CREB)-mediated nuclear transcription and protein synthesis to reduce food intake and lose weight.^325,527^ Besides, increased PKA activity inhibits membrane-bound p-Akt-Ser473 via PI3K/PIP3-mediated translocation of Akt to the membrane, which may promote mTOR/CREB and FoxO signaling pathways.^528^ Second, GLP-1-producing neurons in the NTS directly project to other nucleus associated with food intake, such as mesolimbic reward system (MRS) nuclei that includes VTA and the nucleus accumbens.^529^ Injection of the GLP-1R agonist in rat VTA activates GLP-1R and suppresses food intake through AMPA/kainate receptor signaling.^530^ Likewise, intra-NAc core GLP-1R activation prevents weight gain at least in part via AMPA/kainate receptor signaling.^529^ These findings provide valuable insights into the negative energy balance mediated by GLP-1R signaling engaged glutamatergic neurotransmission in the MRS. Third, researchers unraveled the neuroanatomical and molecular cascades that modulate feeding behavior in the paraventricular nucleus (PVN) of the hypothalamus, which are NTS–to–PVN glucagon neuronal projection and GLP-1R signaling in the PVN. Specifically, GLP-1R signaling first stimulates the PKA pathway, and then phosphorylates the AMPA receptor subunit glutamate receptor 1 (GluR1, also referred to as GluA1) at S845 to enhance GluA1 membrane trafficking, ultimately augmenting excitatory effects on postsynaptic neurons.^531^ Fourth, AP is a circumventricular organ that regulates emesis. Electrophysiological effects of GLP-1 on mice AP neurons indicated that GLP-1 directly binds to Gαs and elicits AC that converts adenosine triphosphate (ATP) to cAMP, resulting in the activation of the AC/cAMP/PKA signaling pathway.^532^

Lipases comprise lingual lipase, gastric lipase, and pancreatic lipase. Lingual lipase has a weak effect on fat degradation, but in infants and young children, it can degrade about 50–70% of ingested fat. It is generally accepted that gastric lipase is a regulator of pancreatic lipase secretion and plays an auxiliary role in lipolysis.^533–535^ Compared with lingual lipase and gastric lipase, the role of pancreatic lipase in lipolysis is self-evident. The pancreatic lipase directly participates in the regulation of intestinal absorption of fatty acids. When the human body ingests dietary fats, gastric lipase and pancreatic lipase hydrolyze about 10–30% and 50–70% of the lipids, respectively, generating substances including monoglycerides and free fatty acids, which are subsequently absorbed by the intestine. Next, monoacylglycerols are resynthesized into triacylglycerols and stored in the form of adipose tissue for energy deposition.^533,536,537^ Being the solitary diet drug targeting lipase currently in clinical use, orlistat is available on the market as two different products, Xenical (Roche) and Alli (GlaxoSmithKline), which are prescription and over-the-counter respectively (Fig. 7).^538^ Orlistat exerts its lipid-inhibiting effect without acting on the CNS or entering the bloodstream.^533^ Specifically, orlistat localizes active site serine residues of human gastric and pancreatic lipases, which form stoichiometric long-lived acyl-enzyme complexes upon nucleophilic attack on their β-lactone rings. This covalent binding impedes the hydrolysis of dietary triacylglycerols, thereby reducing monoglycerides and free fatty acids, and eventually decreasing fat storage and achieving the purpose of weight loss.^533,539–543^

### AOMs under clinical trial

#### GLP-1R agonist

Even with the landmark research results of liraglutide in 2009^544^ and semaglutide in 2021^488^ and their great success in the market, the enthusiasm for exploration of different GLP-1R agonists does not seem to have faded. We listed the clinical trials concerning GLP-1R agonists after 2015 in Table 1, and their mechanisms of action have been detailed previously.

#### GCGR agonist

Glucagon acts through the coupling of glucagon receptor (GCGR) to Gαs and Gq proteins, which trigger the activities of AC and phospholipase C (PLC), respectively.^545^ It is generally believed that AC catalyzes the conversion of ATP to cAMP and PLC hydrolyzes phosphatidylinositol 4,5-bisphosphate to generate the secondary messengers diacylglycerol and inositol 1,4,5-triphosphate (IP_3_). Glucagon participates in lipid metabolism by inhibiting and promoting hepatic lipogenesis and β-oxidation, respectively.^545^ Specifically, glucagon stimulates AC on the adipocyte’s membrane of the liver, leading to increased activity of the cAMP/PKA signaling pathway and phosphorylation/activation of downstream HSL. Phosphorylated HSL converts diglycerides to monoglycerides and yields free fatty acids via monoacylglycerol lipase, thereby attenuating lipogenesis. The activated cAMP/PKA signaling pathway phosphorylates/inactivates acetyl-CoA carboxylase (ACC) to inhibit the conversion of acetyl-CoA to malonyl-CoA, thereby inhibiting the de novo synthesis of fatty acids. Furthermore, the activated cAMP/PKA signaling activates CREB, PPARα, and FoxA2 to induce the transcription of genes required for β-oxidation.^545^ In a recent article published in *Nature*, Perry et al. unraveled an IP_3_ receptor type I (IP_3_R-I/INSP3R1)-dependent signaling pathway of glucagon-induced lipolysis in the liver. They found that INSP3R1 integrates signals from Gαs/cAMP/PKA and Gq/PLC/IP_3_ cascades and releases Ca^2+^ into the mitochondria and cytosol to increase β-oxidation and Ca^2+^/calmodulin (CaM)-dependent protein kinase II/ATGL-mediated lipolysis, respectively.^546,547^ Glucagon enhances FGF21 release in the liver to stimulate thermogenesis in BAT or directly stimulates BAT and browning of WAT to raise energy consumption.^548^ Glucagon induces satiety by regulating the PKA/CaM-dependent protein kinase kinase β (CaMKKβ)/AMPK/AgRP signaling pathway through the liver-brain axis, which triggers satiety signals in the liver and maps to AP and NTS through the hepatic branch of the vagus nerve, and then transmits to the hypothalamic ARC.^549–551^ Long-acting glucagon is more suitable for use in weight loss in that it has an extremely short half-life in rodents.^551,552^ HM15136 is a novel long-acting glucagon analog developed by Hanmi Pharmaceutical^TM^ that treats obesity by regulating liver-targeted signaling pathways, energy expenditure, and satiety (Table 1).^553^

#### GIPR agonist and antagonist

Currently, the field regarding glucose-dependent insulinotropic polypeptide/gastric inhibitory polypeptide (GIP) is still in the phenomenological stage and both positive and negative modulation of GIP receptor (GIPR) activity can lead to weight loss.^554^ This mechanism is backed by admittedly preclinical research and unique among clinical-stage assets. Therefore, there are existing dual agonists in which GLP-1R agonist is combined with GIPR agonist or antagonist. AMG133 is a drug consisting of GLP-1R agonist and GIPR antagonist and is currently under phase 1 clinical trial (Table 1). It might take time to determine whether such a mechanism works in humans.

#### GLP-1R-based dual or triple agonists

GLP-1R agonists such as liraglutide and semaglutide have been in clinical use to treat obesity for nearly 8 years and 1 year, respectively. However, even with great success, it is undeniable that there is still a huge gap in weight management between GLP-1R agonist monotherapy and bariatric surgery. To achieve equivalent efficacy using a noninvasive strategy, researchers have envisioned a “co-agonist” blueprint combining GLP-1R agonists with GCGR agonists or GIPR agonists, or both.^555^ In rodents, combined GLP-1 and glucagon administration increased c-Fos expression in the brainstem and amygdala and exhibited a synergistic effect on food intake reduction.^556^ In humans, coadministration of GLP-1 and glucagon results in a superior reduction in food intake than either GLP-1 or glucagon alone.^557^ Besides, GLP-1 can neutralize hyperglycemia caused by glucagon,^558^ and polypharmacy can achieve the same reduction in food intake or increase in energy expenditure at fewer doses.^559^ Cotadutide (MEDI0832) is the first GLP-1R/GCGR dual agonist under clinical trial and progresses well.^560,561^ By contrast, other dual agonists have shown mixed results.^562^ Efinopegdutide (JNJ-64565111) and liraglutide resulted in placebo-adjusted weight loss of 6.7–10.0 and 5.8%, respectively, and gastrointestinal adverse events occurred in 89 and 60%, respectively.^563^ Besides, the clinical trials of SAR425899 were halted owing to severe gastrointestinal adverse events.^564^ Several other GLP-1R/GCGR dual agonists are currently in development including pemvidutide/ALT-801, BI 456906, CT-388, CT-868, DD01, and NN9277/NNC9204-1177 (Table 1). Similarly, the observation that GLP-1R/GIPR dual agonist enhanced weight loss in mice has successfully set off an upsurge in the study of GLP-1R/GIPR dual agonists.^565,566^ For example, tirzepatide (LY3298176) has been shown to surpass semaglutide in glucose and body weight control.^567^ Therefore, it is rational to step up the pace for the development of GLP-1R/GIPR/GCGR triple agonist. LY3437943 is a novel GLP-1R/GIPR/GCGR triple agonist and showed superior weight loss in mice compared to other incretin receptor-targeting molecules.^568^ Furthermore, Bossart and Konkar developed SAR441255 and found that treatment of the novel peptide triagonist showed greater metabolic outcomes in mice and monkeys (Table 1).^569^

#### Oxyntomodulin (OXM) analog

OXM, as well as GLP-1 and PYY, are intestinal anorectic hormones secreted from the enteroendocrine L cells.^570^ OXM stands for a weak but glucagon-dominant GLP-1R/GCGR dual agonist as it is 3- and 100-fold less potent than the cognate ligands glucagon and GLP-1 concerning cAMP accumulation, respectively.^562,571^ OXM has exhibited stronger efficacy in weight and glucose management compared to pure GLP-1R agonists in several preclinical studies.^572^ Central and peripheral OXM administration can reduce food intake in rodents and humans or rodents, respectively.^573^

#### Dipeptidyl peptidase-4 (DPP-4) inhibitor

Like glucagon, both GLP-1 and GIP have characteristically short circulating half-lives, suggesting that they are rapidly proteolytically hydrolyzed by several peptidases in plasma, leading to restricted therapeutic utility and widespread use.^574,575^ DPP-4 plays a quantitatively pivotal role among these peptidases and its active inhibitors that augments incretin levels by delaying clearance of GLP-1 and GIP are developed to abrogate this pharmacokinetic limitation.^576^ In addition to weight loss by indirectly increasing the expression of GLP-1 and GIP, DPP-4 inhibitors also achieve weight control in other ways. Catalán et al. are the first to find that caveolin-1 (CAV-1), an integral membrane protein most abundantly distributed in adipose tissue,^577^ is upregulated in visceral and subcutaneous adipose tissue in obese patients compared to lean controls, regardless of glucose levels.^578^ They also revealed a significant correlation between CAV-1 mRNA expression and several inflammatory markers.^578^ In adipocytes, CAV-1 modulates insulin transduction via the Akt signaling pathway.^579^ Intriguingly, active site in DPP-4 was indispensable in the interaction between DPP-4 and CAV-1.^580^ These pieces of evidence raise a possible way to beat obesity, that is blocking the interaction between DPP-4 and CAV-1 to improve adipocyte insulin sensitivity. The burning of glucose and fat in brown and beige adipose cells for heat production is primarily mediated by UCP1.^581^ Takeda et al. deciphered that the DPP-4 inhibitor upregulates UCP1 expression via the inhibition of the ERK1/2 signaling pathway, indicating that long-term use of the DPP-4 inhibitor could significantly improve body weight and energy homeostasis by modulating BAT activity and is a possible option to cure obesity.^582^

#### Amylin receptor (AMYR) and calcitonin receptor (CTR) agonists

Amylin has been reported to inhibit gastric emptying through specific binding to AMYR in the gastric fundus and mapping of corresponding neuronal signals to AP and NAc in the hindbrain.^583^ Amylin induces an anorectic effect based on its positive stimuli on AP neurons through cGMP, c-Fos, and ERK1/2 signaling pathways.^584–586^ Amylin also induces anorexia by increasing brain 5-HT, stimulating histamine H1 and dopamine D2 receptors, and inhibiting NPY-induced feeding.^587–590^ Amylin serves as one of the few molecules owning the ability to restore leptin sensitivity in diet-induced obesity by potentiating leptin-induced p-STAT3 in ARC and VMN of the hypothalamus.^591,592^ Moreover, amylin increases IL-6 to enhance the activation of leptin-induced p-STAT3 in the VMN.^593^ The human AMYR isoforms are CTR-based complexes incorporating receptor activity-modifying proteins.^594,595^ Studies have shown that calcitonin induces signaling pathways similar to those of amylin in the hindbrain.^594^

#### PYY/NPY receptor type 2 (Y2R) signaling

PYY is co-secreted with GLP-1 and OXM from L cells as PYY_1-36_ and hereupon rapidly converted to its predominant active form PYY_3–36_ by cleavage mediated by DPP-4. PYY_3–36_ is a high-affinity Y2R agonist.^432^ It is demonstrated that postprandial elevation of PYY_3-36_ inhibits food intake and reduces weight gain through PYY/Y2R signaling on both AgRP/NPY and POMC neurons in a gut–hypothalamic projection manner.^596^

#### Ghrelin signaling

Ghrelin is an endogenous ligand of the growth hormone secretagogue receptor type 1a (GHS-R1a).^597^ Prior to secretion into the bloodstream, ghrelin requires post-translational serine octanoylation/acylation by ghrelin O-acyltransferase (GOAT) to bind and activate the GHS-R1a signaling for its orexigenic actions. Human genetic studies have identified that rare mutations and single-nucleotide polymorphisms of GHSR gene might be associated with obesity.^598^ In circulation, esterases can remove the octanoyl group of acylated ghrelin and switch it to unacylated ghrelin. Intriguingly, membrane-anchored GOAT can reacylate unacylated ghrelin to acylated one, which can still function through GHS-R1a signaling.^599–601^ In the VMN of the hypothalamus, the ghrelin-GHS-R1a axis activates AMPK via PLC/IP_3_/Ca^2+^/CaMKKβ and Sirt1/p53 signaling pathways.^599,600^ Besides, CB1 is required for ghrelin to activate AMPK. Activated AMPK inhibits ACC, leading to reduced malonyl-CoA and subsequent carnitine palmitoyltransferase 1A (CPT1A) and 1C (CPT1C) accumulation. AMPK-CPT1A-uncoupling protein 2 and AMPK-CPT1C-ceramide axes potentiate glutamate release from the presynaptic terminals onto AgRP/NPY neurons in ARC and are essential mediators of the effect of ghrelin on feeding. In the ARC of the hypothalamus, the ghrelin upregulates GHS-R1a/mTORC1/S6K and κ-opioid receptor signaling pathways. These two cascades, as well as the effect of glutamate on AgRP/NPY neurons, upregulate key transcription factors pCREB, FoxO1, and BSX to increase mRNA expressions of AgRP and NPY and induce feeding.^602–605^

#### SGLT1/2 inhibitor

There are two sodium-glucose co-transporters (SGLTs) that reabsorb renal glucose, with SGLT2 responsible for more than 90% and the remaining 10% by SGLT1.^606^ SGLT2 inhibitors directly reduce whole body weight by enabling energy expenditure through glucose excretion.^607^ It was observed that SGLT2 inhibition resulted in a significant energy loss of approximately 75 g glucose per day (300 kcal/day).^608^ Besides, osmotic diuresis (107–470 ml/day) brought by SGLT2 inhibitor dapagliflozin in drug-naive patients with T2D may contribute to some weight loss.^609,610^ SGLT2 inhibition activates AMPK signaling and phosphorylates/inactivates ACC to decrease malonyl-CoA, thereby inhibiting the fatty acid synthesis and enhancing β-oxidation.^611–613^ SGLT2 inhibition also motivates adipose thermogenesis and lipolysis via the β-adrenoceptor/cAMP/PKA signaling pathway.^614^ Besides, downregulated SGLT2 promotes the browning of WAT by polarizing M2 adipose tissue macrophages and increasing adiponectin expression in WAT and FGF21 expression in the liver and circulation.^613^ However, the magnitude of weight loss by SGLT2 inhibitors is modest with an average of 1.5–2 kg (placebo-adjusted). The combined administration of SGLT2 inhibitors and anorectics represents a promising way to lose weight.^607^

#### FGF21/FGF receptor 1c isoform (FGFR1c)/β-Klotho signaling

The FGFs form a family of 22 members that regulate a plethora of biological processes including growth, differentiation, development, and metabolism.^615^ Most FGFs function locally as autocrine or paracrine factors, whilst the endocrine FGFs including FGF15/19, FGF21, and FGF23 possess the ability to enter the circulation and function as hormones.^616^ Among them, FGF15/19 and FGF21 hold tremendous potential for medicinal purposes in counteracting obesity because they are important in metabolic regulation.^616,617^ An in vivo study revealed that systemic administration of FGF21 lowered mice body weight by 20%.^618^ FGF21 is a fasting-induced pleiotropic hormone holding pivotal roles in energy balance and glucose and lipid homeostasis via triggering a heterodimeric receptor complex assembled by FGFR1c and β-Klotho.^619^ FGF21 activates the FGFR1c/β-Klotho complex on the membrane of adipocytes, triggering MAPK/mTORC1/S6K signaling and subsequent adiponectin secretion and UCP1 upregulation, improving insulin sensitivity and body weight.^620^ Several lines of evidence have proven that FGF21 administration promotes energy expenditure and losses weight through PGC-1α and CCL11 mediated WAT browning^621^ and central thermogenic hormones-mediated BAT activation.^622^ Furthermore, central infusion of FGF21 in lean rats activates the hypothalamic-pituitary-thyroid axis to induce UCP1 expression in WAT, leading to weight loss.^623^ In contrast, central infusion of FGF21 in obese rats failed to reduce body weight.^624^ Undoubtedly, such a phenomenon and the notion that obesity is an FGF21-resistant state attract us. Emerging in vivo evidence has monitored a dampened ERK1/2/Elk-1/SRF signaling response induced by FGF21/FGFR1c/β-Klotho signaling.^625,626^ Therefore, a deeper investigation of the molecular mechanism whereby obesity impairs FGF21/FGFR1c/β-Klotho signaling may offer novel insights for the FGF21-based obesity drug development.

#### GDF15 agonist

GDF15 is a distant member of the TGF-β superfamily.^627^ Circulating levels of GDF15 are at a low concentration ranging from 0.1 to 1.2 ng/ml under normal physiological conditions. Once the human body is exposed to stress caused by diseases such as tissue damage, cancer, metabolism, and inflammation, the circulation concentration of GDF15 rises by 10- to 100-fold.^628^ Surprisingly, obese individuals have significantly elevated serum GDF15 levels compared to healthy controls,^629^ which may be caused by the liver.^185^ In 2007, four independent studies uncovered that in neurons in AP and NTS, GDF15 binds to GFRAL and then recruits RET, forming a GDF15-GFRAL-RET trimer that induces the phosphorylation of ERK1/2, Akt, and PLC.^630–633^ In the ARC, GDF15 suppresses appetite via upregulating p-ERK1/2 and p-STAT3 and reducing and increasing the mRNA level of NPY and POMC, respectively.^183,185^

## Perspectives

From mechanistic evidence to clinical observations, the causal link between obesity and morbidity/mortality has long been established. Although considerable progress has been made in the understanding of the etiology and pathophysiology of obesity, our evolving knowledge about obesity pathogenesis and personalized therapies are not satisfactory yet. The prescription of moving more and eating less for tackling obesity has now been proven as a crude oversimplification of this complex disease.^634^ Decoding of cellular signaling networks enables us to move towards more precise medicine, enriching our arsenal in the fight against obesity. Indeed, precision therapy can be achieved by targeting specific signals/pathways in different obese populations. Of note, this personalized treatment strategy can be largely enhanced with the help of high-performance computing and artificial intelligence, based on the growing clinical and biological datasets. Nevertheless, owing to the complexity of signaling transductions, identifying the molecular culprits of individual patients is still challenging, which may hamper translation to clinical practice. The success of semaglutide has established a solid foundation for the development of GLP-1R agonists. However, there are more questions than answers. Of primary interest is why GLP-1R agonist works so well, and why there is a huge difference between liraglutide and semaglutide concerning weight control,^487^ which are both GLP-1R agonists. The difference is difficult to attribute to the molecular basis, a situation that seems to exemplify our relatively primitive understanding of the bridge between in vivo efficacy and mechanisms. In addition, we should break the inherent mindset and focus on accelerating the development of energy-consuming drugs, on the basis of understanding the importance of balancing energy intake and energy expenditure. Weight-loss surgery remains the best option for severely obese patients. In this regard, there exists a brilliant future to decipher the signals or pathways involved in obesity through bariatric surgery. In all, the path to seek and develop AOMs remains challenging, and the in-depth learning of known signals and the development and utilization of efficient tools, such as artificial intelligence, are important parts of achieving precision obesity treatment in the future.
