# Correction To: Signaling pathways in obesity: mechanisms and therapeutic interventions

**DOI:** 10.1038/s41392-022-01188-4

**Published:** 2022-10-21

**Authors:** Xue Wen, Bohan Zhang, Beiyi Wu, Haitao Xiao, Zehua Li, Ruoyu Li, Xuewen Xu, Tao Li

**Affiliations:** 1 Department of Plastic and Burn Surgery, National Clinical Research Center for Geriatrics, West China Hospital of Sichuan University, Chengdu, 610041 China; 2 Laboratory of Mitochondria and Metabolism, West China Hospital of Sichuan University, Chengdu, 610041 China; 3 Department of Anesthesiology, National-Local Joint Engineering Research Centre of Translational Medicine of Anesthesiology, West China Hospital of Sichuan University, Chengdu, 610041 China

**Keywords:** Metabolic disorders, Molecular medicine

Correction to: *Signal Transduction and Targeted Therapy* (2022) **7**:298; 10.1038/s41392-022-01149-x, published online 28 August 2022

After online publication of the review,^1^ the authors noticed the order of Figs. 3 to 6 was incorrect. The figures should have appeared as shown below. The original review has been corrected.

**Fig. 3:**
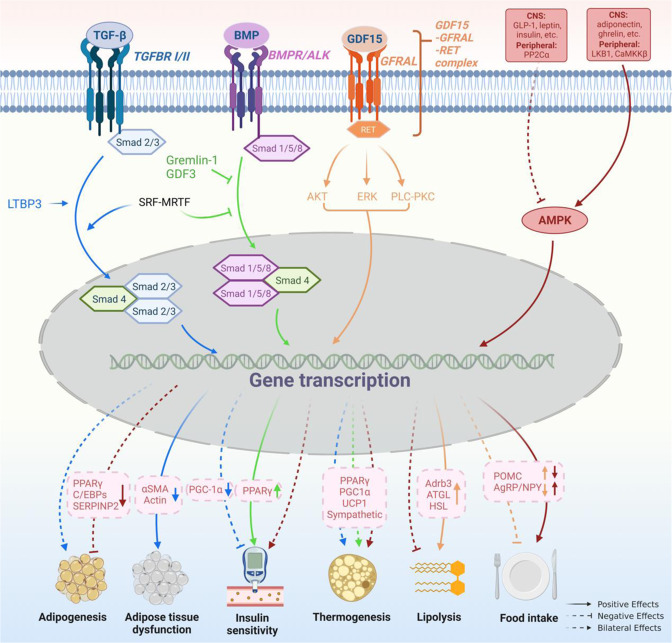
TGF-β and AMPK signaling pathways in obesity pathogenesis. The TGF-β superfamily consists of TGF-β1-3, GDFs, BMPs, etc., which play a diverse role in the development of obesity. TGF-β shows dual effects on adipogenesis/adipocyte differentiation. TGF-β inhibits MSC adipocyte commitment by phosphorylating and suppressing PPARγ and C/EBPs expression, through Smad3 signaling. However, pulsed TGF-β1 administration during the commitment phase shows a promotion effect on adipogenesis in MSC by down-regulating SERPINB2 expression. In adipocytes, TGF-β signaling is involved in adipose tissue dysfunction by enhancing the expression of myofibroblast signature genes. The role of TGF-β in BAT-associated thermogenesis is also controversial. Activation of TGF-β signaling by LTBP3 promotes WAT browning by modulating UCP1 expression, while hepatic TGF-β signaling contributes to HFD-induced steatosis and obesity by reducing mitochondrial respiration and inhibiting white-to-beige fat conversion. In addition, SRF - MRTF - axis which transcriptionally enhances the TGF-β but attenuates BMP signaling pathway suppresses brown adipogenesis. TGF-β/Smad3 signaling also plays a negative role in insulin sensitivity by suppressing PGC-1α expression in adipose tissue. BMP seems to play a contrary role to TGF- β in the regulation of insulin sensitivity by up-regulating PPARγ expression. Similar to TGF- β, the role of BMP in BAT-associated thermogenesis is inconsistent. BMP4 promotes WAT browning and this process is inhibited by Gremlin-1. However, BMP-4 signaling during the terminal differentiation phase can impair the acquisition of a mature brown adipocyte phenotype. GDF15, another member of TGF-β superfamily, was identified as a potential target for the treatment of obesity. By interacting with GFRAL and followed by the activation of AKT-, ERK-, and PLC-PKC signaling pathway, GDF15 stimulates lipolysis by up-regulating Adrb3, ATGL, and HSL expressions. It also inhibits food intake in a CNS-dependent manner via an unknown mechanism. AMPK is a heterotrimer complex. It is activated by adiponectin, ghrelin, etc. in CNS and LKB1 and CaMKKβ in peripheral tissue, and inactivated by GLP-1, leptin, etc. in CNS and PP2Cα in peripheral tissue. Activation of AMPK in CNS results in hyperphagia, insulin resistance, decreased thermogenesis, and weight gain. While, in adipocytes, it results in inhibited adipogenesis, insulin sensitiveness, enhanced thermogenesis, and weight loss. However, AMPK limits lipolysis since AMPK is an enzyme in case of energy shortage

**Fig. 4:**
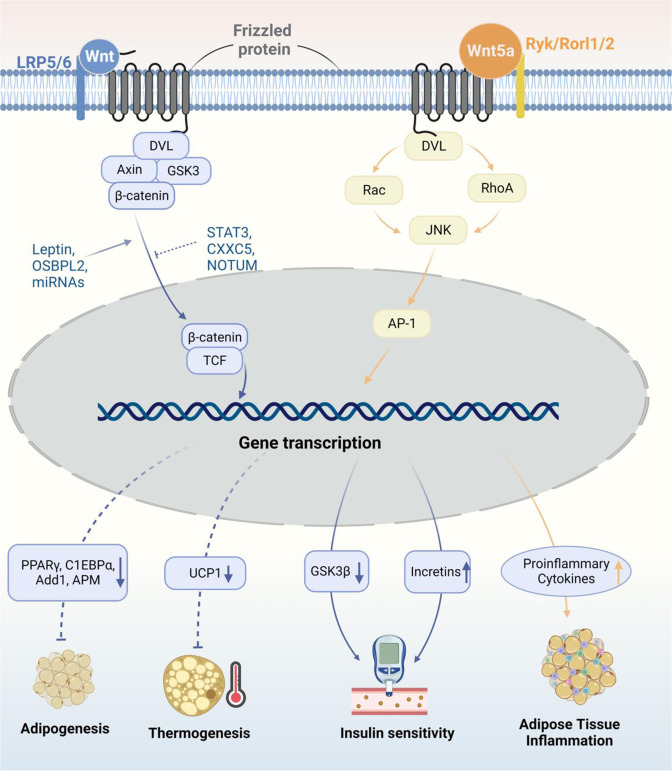
Wnt/β-catenin pathways in obesity pathogenesis. In the canonical Wnt pathway, upon activation by Wnt proteins, β-catenin is released and enters the nucleus as a transcription coactivator of TCF to regulate the transcription of target genes. The activation of Wnt/β-catenin pathway leads to, (1) the supersession of adipogenesis by down-regulating the expression of PPARγ, C1EBPα, Add1, APM, etc.; (2) the inhibition of BAT-related thermogenesis by down-regulating UCP-1; and (3) the increase of insulin sensitivity by down-regulating GSK3β expression in CNS while up-regulating incretins within the small intestinal epithelium. The canonical Wnt signaling can be stimulated by factors including leptin, OSBPL2, and miRNAs like miR-23b, miR-148b miR-4269, and miR-4429. It can also be inhibited by JAK/STAT3 pathway, CXXC5, and NOTUM. These factors are all involved in the pathogenesis of obesity by regulating Wnt/β-catenin signaling pathway. Additionally, Wnt5a, a part of the non-canonical Wnt pathway, induces obesity-associated inflammation in WAT in a JNK-dependent manner, which further contributes to the occurrence of insulin resistance in adipose tissue

**Fig. 5:**
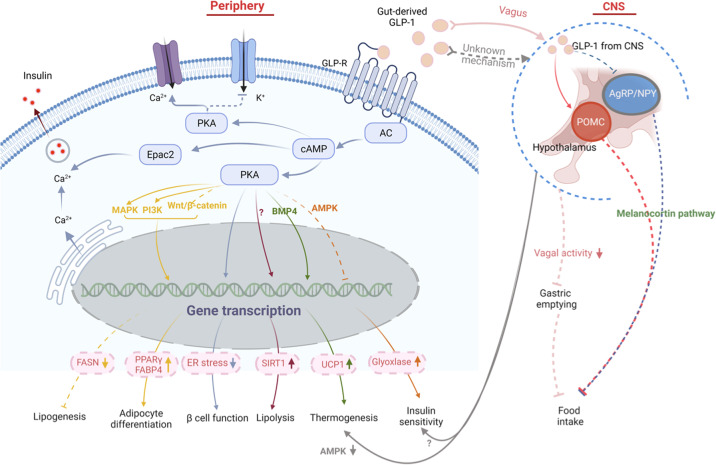
GLP-1 signaling pathway in obesity pathogenesis. The anti-obesity effect of GLP-1 can be mediated by either peripheral or central signals. In the periphery, the activation of GLP-R by gut-derived GLP-1 enhances the glucose-stimulated insulin secretion, through PKA-dependent or Epac2 pathway. By enhanced PKA activity, GLP-1 alleviates insulin resistance and leads to weight loss in obese diabetic mice by reducing ER stress and improving β-cell function. It also improves insulin sensitivity in peripheral tissue by suppressing AMPK-related pathway and elevating glyoxalase. By interacting with multiple signaling pathways including PI3K, MAPK, and Wnt4-β-catenin pathways, GLP-1 promotes pre-adipocyte differentiation by up-regulating PPARγ and FABP4, but suppresses lipogenesis in mature adipocytes by decreasing fatty acid synthase expression. GLP-1 also enhances lipolysis in WAT by increasing the expression and activity of Sirt1, through yet unknown mechanisms. Additionally, GLP-1 participates in the regulation of thermogenesis by inhibiting BMP4-related signaling pathway and thus induces the expression of thermogenic genes like UCP1. Gut-derived GLP-1 also interacts with GLP-R expressed in vagus, through which the information is transmitted upward to the CNS, which in turns suppresses vagal activity and gastric emptying, so as to increase satiety and reduce food intake. Besides, peripheral GLP-1 plays a role in the regulation of insulin sensitivity and BAT-related thermogenesis in a CNS-dependent manner. the latter is partially mediated by suppressing AMPK signaling pathway. Central GLP-1 produced by neurons in the caudal medulla is transmitted into the hypothalamus and functions to reduce food intake by activating POMC neurons while suppressing AgRP/NPY neurons in this area

**Fig. 6:**
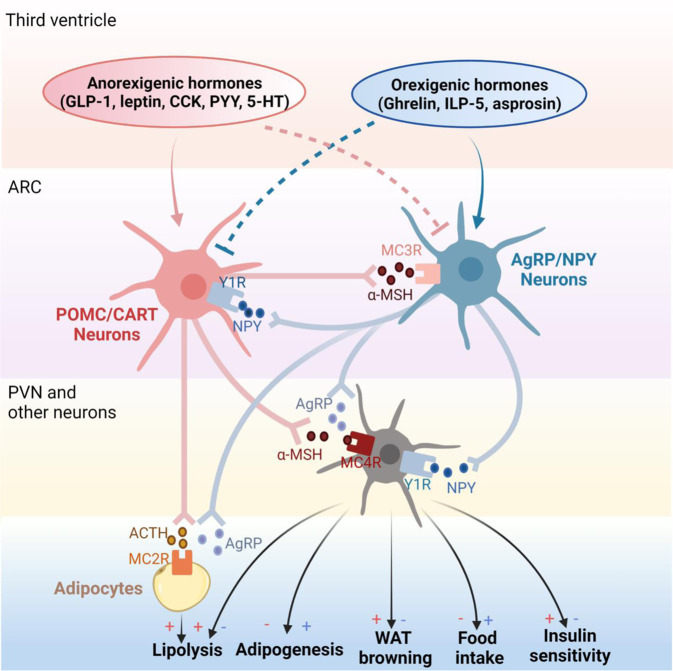
Melanocortin pathway in obesity pathogenesis. The melanocortin pathway consists of POMC; melanocortin receptors MC1R-MC5R; and agouti and AgRP. POMC/CART neurons in ARC are stimulated by anorexigenic hormones in the third ventricle like GLP-1, leptin, CCK, PYY, and 5-HT, while suppressed by orexigenic hormones like Ghrelin, ILP-5, and asprosin. Upon stimulation, POMC/CART neurons secrete POMC including α-MSH and ACTH. α-MSH is released into the PVN. By interacting with MC4R, α-MSH activates PVN neurons and displays anti-obesity effects by inhibiting adipogenesis, promoting lipolysis, inducing WAT browning, reducing food intake, and improving insulin sensitivity. ACTH released by POMC/CART neurons actions on adipocytes directly by binding to MC2R, further promoting lipolysis. However, these effects can be abolished by AgRP, which is the endogenous antagonist of POMC and is secreted by AgRP/NPY neurons in ARC. Conversely, AgRP/NPY neurons can be stimulated by orexigenic hormones in the third ventricle but inhibited by anorexigenic hormones. Notably, POMC/CART and AgRP/NPY neurons interact mutually. NPY receptor Y1R is expressed in POMC/CART neurons and its activation inhibits POMC neurons in the ARC. In contrast, MC3R expressed in AgRP/NPY neurons seems to increase food intake in an “AgRP circuitry”-dependent manner
